# Cell-type-specific tuning of Cav1.3 Ca^2+^-channels by a C-terminal automodulatory domain

**DOI:** 10.3389/fncel.2015.00309

**Published:** 2015-08-24

**Authors:** Anja Scharinger, Stephanie Eckrich, David H. Vandael, Kai Schönig, Alexandra Koschak, Dietmar Hecker, Gurjot Kaur, Amy Lee, Anupam Sah, Dusan Bartsch, Bruno Benedetti, Andreas Lieb, Bernhard Schick, Nicolas Singewald, Martina J. Sinnegger-Brauns, Emilio Carbone, Jutta Engel, Jörg Striessnig

**Affiliations:** ^1^Department of Pharmacology and Toxicology, Institute of Pharmacy, Center for Molecular Biosciences, University of InnsbruckInnsbruck, Austria; ^2^Department of Biophysics, Center for Integrative Physiology and Molecular Medicine, Saarland UniversityHomburg, Germany; ^3^Laboratory of Cellular and Molecular Neuroscience, Department of Drug Science, Nanostructured Interfaces and Surfaces Center, University of TorinoTorino, Italy; ^4^Department of Molecular Biology, Central Institute of Mental Health, Medical Faculty Mannheim, Heidelberg UniversityMannheim, Germany; ^5^Department of Otorhinolaryngology, Saarland UniversityHomburg, Germany; ^6^Department of Molecular Physiology and Biophysics, University of IowaIowa City, IA, USA; ^7^Department of Physiology and Medical Physics, Innsbruck Medical UniversityInnsbruck, Austria

**Keywords:** calcium channels, channel gating, hearing, hair cell, alternative splicing, chromaffin cells

## Abstract

Cav1.3 L-type Ca^2+^-channel function is regulated by a C-terminal automodulatory domain (CTM). It affects channel binding of calmodulin and thereby tunes channel activity by interfering with Ca^2+^- and voltage-dependent gating. Alternative splicing generates short C-terminal channel variants lacking the CTM resulting in enhanced Ca^2+^-dependent inactivation and stronger voltage-sensitivity upon heterologous expression. However, the role of this modulatory domain for channel function in its native environment is unkown. To determine its functional significance *in vivo*, we interrupted the CTM with a hemagglutinin tag in mutant mice (Cav1.3DCRD^HA/HA^). Using these mice we provide biochemical evidence for the existence of long (CTM-containing) and short (CTM-deficient) Cav1.3 α1-subunits in brain. The long (HA-labeled) Cav1.3 isoform was present in all ribbon synapses of cochlear inner hair cells. CTM-elimination impaired Ca^2+^-dependent inactivation of Ca^2+^-currents in hair cells but increased it in chromaffin cells, resulting in hyperpolarized resting potentials and reduced pacemaking. CTM disruption did not affect hearing thresholds. We show that the modulatory function of the CTM is affected by its native environment in different cells and thus occurs in a cell-type specific manner *in vivo*. It stabilizes gating properties of Cav1.3 channels required for normal electrical excitability.

## Introduction

Ca^2+^ entering cells through voltage-gated L-type Ca^2+^-channels (LTCCs, Cav1) serves as important second messenger for many different cellular events. It is required for normal muscle contraction, hormone secretion, sensory cell signaling, neuronal excitability and plasticity (Simms and Zamponi, 2014; Striessnig et al., 2014 for reviews). Four α1-subunits (Cav1.1–Cav1.4) form different LTCC isoforms. Cav1.1 (excitation-contraction coupling in skeletal muscle) and Cav1.4 (retinal signaling) serve more restricted functions, whereas Cav1.2 and Cav1.3 are widely expressed in the mammalian organism and often co-exist in the same cell (Berger and Bartsch, 2014; Striessnig et al., 2014). Different biophysical properties and protein interactions allow them to support distinct physiological processes (Shaw and Colecraft, 2013; Simms and Zamponi, 2014; Striessnig et al., 2014). In the brain, Cav1.2 and Cav1.3 are located at postsynaptic sites where their Ca^2+^ signals couple synaptic activity to gene transcription (Ma et al., 2013) and thus play a key role in synaptic plasticity, different types of memory and neuronal development (Striessnig and Koschak, 2008; Simms and Zamponi, 2014; Striessnig et al., 2014). Cav1.3 also stabilizes upstate potentials, regulates spine density and synaptic refinement (Simms and Zamponi, 2014; Striessnig et al., 2014). In cochlear inner hair cells (IHCs), Cav1.3-mediated Ca^2+^ influx drives sound-induced glutamate release and is essential for hearing. Cav1.3 supports normal sinoatrial node pacemaking. Cav1.3-deficient (Cav1.3^-/-^) mice are deaf and show sinoatrial node dysfunction (Simms and Zamponi, 2014; Striessnig et al., 2014). The low activation threshold of Cav1.3 channels (Lipscombe et al., 2004; Lieb et al., 2014; Striessnig et al., 2014) enables them to contribute to pacemaking also in adrenal chromaffin cells (Marcantoni et al., 2010; Vandael et al., 2012). Moreover, Cav1.3 provides Ca^2+^ for aldosterone production (Azizan et al., 2013; Scholl et al., 2013).

Human diseases resulting from aberrant Cav1.3 LTCC function (CACNA1D gene) have been described. Cav1.3-deficiency replicates the phenotype observed in mice with sinoatrial node dysfunction and deafness (SANDD, OMIM #614896; Baig et al., 2011). In contrast, CACNA1D mutations that alter Cav1.3 gating properties leading to enhanced Ca^2+^ currents affect also other tissues, including the brain. Mutations enhancing Cav1.3 activity were discovered in patients with a severe congenital multiorgan syndrome with primary aldosteronism, seizures and neurologic abnormalities including global developmental delay and intellectual disability (PASNA, OMIM #615474) (Azizan et al., 2013; Scholl et al., 2013). Moreover, we have recently reported that similar *de novo* CANCNA1D mutations strongly contribute to disease risk in two patients with autism and intellectual impairment (Pinggera et al., 2015). These findings illustrate the importance for tight control of Cav1.3 activity, and that dysregulation of Cav1.3 predisposes to neuropsychiatric and neurodevelopmental disorders (De Rubeis et al., 2014; Striessnig et al., 2015).

Considering the essential physiological roles of LTCCs, an important question is how channel function is adjusted *in vivo* to prevent inappropriate Ca^2+^ signals. One well-characterized autoinhibitory mechanism inherent to most VGCCs is Ca^2+^-induced inactivation (CDI), which limits Ca^2+^ influx in response to Ca^2+^ entry and toxic intracellular Ca^2+^ accumulation (for recent review Ben-Johny and Yue, 2014). Calmodulin (CaM) binding to the proximal C-terminus of the pore-forming α1-subunit mediates the Ca^2+^-induced conformational changes promoting CDI (Ben-Johny and Yue, 2014). However, CDI itself is further subject to fine-tuning. In the cochlea CaM-mediated CDI is strongly suppressed by competing Ca^2+^-binding proteins (CaBPs) that do not support CDI (Cui et al., 2007; Schrauwen et al., 2012; Ben-Johny and Yue, 2014). In the case of Cav1.3, two other mechanisms have been identified that can reduce CaM affinity for the C-terminus and thus CDI: RNA-editing (Huang et al., 2012; Bazzazi et al., 2013) and a C-terminal automodulatory domain (CTM; Singh et al., 2008; Tan et al., 2011).

This CTM forms by interaction of two putative α-helical domains – a proximal and a distal C-terminal regulatory domain (PCRD and DCRD, respectively; Singh et al., 2008). In brain and other tissues, alternative splicing of Cav1.3 α1 generates C-terminally truncated Cav1.3 α1 mRNA species that lack a functional CTM, i.e., C-terminally long and short Cav1.3 α1 isoforms (Bock et al., 2011; Tan et al., 2011). Biochemical and functional studies in HEK-293 cells revealed that the CTM forms a module that inhibits CDI by competing with CaM binding to its well characterized interaction sites within the proximal C-terminal tail (Ben-Johny and Yue, 2014) and that it also decreases channel open probability and reduces the voltage-sensitivity of pore opening (Singh et al., 2008; Bock et al., 2011; Lieb et al., 2014). Therefore alternative splicing affects Cav1.3 channel activity. Despite these detailed studies in recombinant systems the role of this modulatory mechanism for *in vivo* channel function is completely unknown. Although two size forms of Cav1.3 α1 have been detected in rodent brain (Hell et al., 1993) unequivocal proof for the existence of C-terminally short forms without functional CTM is lacking. It is also unclear whether these different size forms arise from alternative splicing or from C-terminal proteolytic processing as reported for Cav1.2 (Gomez-Ospina et al., 2006; Hulme et al., 2006). Although functional studies with recombinant channels predict enhanced CDI, higher open probability, and channel activation at lower voltages for short splice variants *in vitro*, the physiological significance of this splicing-dependent regulation of Cav1.3 channel gating *in vivo* is still unclear. It is also difficult to predict how the native cellular environment affects Cav1.3 regulation by the CTM. For example, it is unclear if the CTM also affects channel function in cells in which CaBPs strongly compete with CaM and largely remove CDI.

To address this question we generated a novel mouse model in which we disrupted CTM function in the long Cav1.3 C-terminus by replacing part of the DCRD domain in exon 49 of the CACNA1D gene by homologous recombination with an HA-epitope (Cav1.3DCRD^HA/HA^ mice). This allowed us to directly immunolabel CTM-containing Cav1.3 variants and to quantify the functional consequences of disrupted CTM function *in vitro* and *in vivo*. We provide biochemical evidence for the existence of long and short Cav1.3 α1-subunit polypeptides with and without CTM, identify the long variants as intrinsic constituent of all ribbon synapses in IHCs and discovered an unexpected, cell-specific regulation of Cav1.3 CDI in mouse chromaffin cells (MCCs) and IHCs. We further show that the CTM controls resting membrane potential and spontaneous pacemaking in MCCs. Our data reveal the CTM as an important regulatory mechanism required for normal Ca^2+^ signaling.

## Materials and Methods

All procedures with animals were approved by the national ethical committee on animal care and use (Austrian Bundesministerium für Wissenschaft und Forschung) and are in compliance with international laws and policies.

### Cloning of cDNA Constructs

mCav1.3_L_-HA: mCav1.3_L_–HA was generated by replacing amino acids 2080-2083 (DEME) in the DCRD region of the mouse Cav1.3 α1 subunit cDNA (Klugbauer et al., 2002; Genbank accession NM_001083616) by an HA-antibody tag. Correct integration of the HA-tag was verified by sequencing (Eurofins MWG Operon).

### Generation of Cav1.3DCRD^HA/HA^ Mice

Bac clone bMQ427c09 (Adams et al., 2005), which includes exon 8–49 of the mouse CACNA1D gene was digested with BamHI, and a 6399 bp fragment containing exon 48 and 49 (position 30045040-30038642, Ref: NC_000080.6) was ligated with BamHI-digested and dephosphorylated pBluescript II SK (-) (pBS). The resulting subclone (BampBS) was digested with NaeI and HindIII and religated after blunting with T4 DNA Polymerase (Fermentas) to exclude a ClaI restriction site. For extension of the genomic region, a PCR product with an additional artificial NotI^∗^ restriction site was amplified (primer: fwd: 5′-ATA ATA GCG GCC GCT GAG CTT ATG TCC CCA ATT AG-3′; rev, 5′-GCT GGG GTG CAC TAC CCA CT-3′, template bMQ427c09, position 30037757 – 30039650 of NC_000080.6), subcloned into pJET1.2 (Thermo Scientific, Germany) and transferred into BampBS via BstEII – NotI (1987 bp) fragment exchange, yielding BamExtpBS. A 279 bp ClaI-BsmBI fragment resembling position 30042386 – 30042050 in NC_000080.6 was synthesized (Eurofins MWG Operon). The synthetic fragment contained an HA-tag to replace amino acid residues DEME (single amino acid letter code; 30042270 – 30042259 in NC_000080.6) in the DCRD domain (Singh et al., 2008) and artificial restriction sites XbaI^∗^ and SalI^∗^ after the stop codon. The synthetic fragment was demethylated by transformation of Dam^-^ and Dcm^-^ *Escherichia coli* GM 2163 (Thermo Scientific, Germany) and incorporated into BamExtpBS via ClaI – BsmBI fragment exchange, yielding BamExtSynpBS. The neomycin resistance gene was removed from pL452 (ncifrederick.cancer.gov; Liu et al., 2003) by XhoI digest and ligated with the SalI – digested and dephosphorylated BamExtSynpBS construct, yielding BamExtSynNeopBS. The negative selection marker HSV-TK was amplified by PCR (primers: fwd, 5′-CTC GAG GCT AGA ACT AGT GG-3′; rev, 5′-GGT ATC GAC AGA GTG CCA G-3′, template: pL253 (ncifrederick.cancer.gov; Liu et al., 2003) and incorporated into the SmaI digested BamExtSynNeopBS construct via blunt end ligation. The construct sequence was verified by Eurofins MWG operon. Mutant mice were generated using standard procedures for homologous recombination in ES-cells (R1 derived from 129/Sv). Clones with correct targeting were identified by PCR with the longAmp Taq DNA Polymerase (New England Biolabs). Primers for short homologous arm: FH1, 5′-GTC CTT CCA TCG CCT GCC CTG CCT C-3′; RH1, 5′-TCG ACG ACC TGC AGC CAA GCT AGC T-3′; primers for long homologous arm: FH2, 5′-GCT TTA CGG TAT CGC CGC TCC CGA TTC G-3′; RH2, 5′-CCC CTG GCT GCC TGC GGG TAG C-3′. In positive clones selected for blastocyst (C57BL/6) injection the integrity of recombined sequence was verified by sequencing. Chimeric males were paired with C57BL/6 mice. Heterozygous offspring were paired with mice expressing Cre recombinase [TgN(EIIa-Cre)C5379Lmgd, H. Westphal, NIH Bethesda, USA] to remove the neo selection marker. Resulting heterozygous offspring were interbred to obtain homozygous mutants (Cav1.3DCRD^HA/HA^) as well as wild-type (WT) littermates. Age-matched littermates were used for all experiments. For genotyping the following PCR primers were used (**Figure 2**): forward F1: 5′-TCT GTG CTA CGT CCC CAG TGC T-3′; reverse: R1: 5′-GCA GCA CTA GCG TAA TCT GGA ACA T-3′; R2: 5′-CGT GCC CGT CTC TGG CTG GA-3′ (WT allele: 535 bp, mutated allele: 332 bp + 678 bp).

#### RNA Isolation, Reverse Transcription and Qualitative PCR Analysis in IHCs and OHCs

RNA and cDNA samples were obtained from adult male mouse IHCs and OHCs. Reverse transcription of 40 individually collected IHCs or 120 OHCs in a reaction volume of 20 μl was carried out with the SuperScript III Reverse Transcriptase (Fermentas), dNTP (New England Biolabs), random hexamer primers (Invitrogen), RNaseOUT (Invitrogen), dithiothreitol and nuclease free water (Promega). PCR (94°C for 1 min, 40 cycles of 94°C for 30 s, 58°C for 30 s, 72°C for 1 min) was performed with 3 μl of the reverse transcription product in a reaction volume of 25 μl with the PCR Master Mix (2x) (Fermentas) and 0.4 μM primer. The following primers within exon 42 and exon 45 were used to amplify a 624 bp stretch for transcripts containing exon 43L or 470 bp for exon 43S (fwd: 5′-GGG CCA GAA ATC CGA CGG GC-3′; rev: 5′-TCC AGG TGG GAG AGC TGT CGT-3′). To obtain detectable PCR products (43L: 557 bp; 43S: 403 bp) a second (nested) PCR (25 cycles, same program) with 0.2 μl of the first PCR product as template with exon 42 and 45-specific primers was necessary (fwd: 5′-ACG AGC CAG AAG ACT CCA AA-3′; rev: 5′-CAC AGC ACT CCT CGC TAC TG-3′). 0.15 ng RNA equivalent of whole brain and of whole heart cDNA served as positive controls.

### Primary Antibodies

Anti-HA, high affinity rat monoclonal antibody (3F10, Roche, 1:200) or Alexa488-conjugated antibody (mouse, Invitrogen); anti-Cav1.3α1_CT,_ affinity-purified polyclonal antibody directed against amino acids 2022-2138 (GenBank accession M76558; Platzer et al., 2000); anti-Cav1.3α1_NT_ (Ab144, Jenkins et al., 2010); anti-CtBP2/RIBEYE (rabbit, Cell Applications); anti-Cav1.3 (rabbit, Alomone); anti-Cavβ2 (rabbit, kindly provided by V. Flockerzi, Saarland University).

### Protein Preparations from Transfected tsA-201 Cells and Mouse Whole Brain

For preparation of membranes medium was removed 3 days after transfection and cells were washed with ice-cold PBS (phosphate buffered saline, 137 mM NaCl, 2.7 mM KCl, 8 mM Na_2_HPO_4_ × 2H_2_0, 1.5 mM KH_2_PO_4,_) and harvested by scraping. The cells were resuspended in 2 ml lysis buffer (10 mM Tris-HCl, 1 μg/ml aprotinin, 0.1 mg/ml trypsin inhibitor, 1 μM pepstatin A, 0.5 mM benzamidine, 0.2 mM PMSF (phenylmethylsulfonyl fluoride), 2 mM iodacetamide, 1 μl/ml leupeptin) and kept on ice for 15 min. Cells were resuspended by pipetting up and down 50 times and subsequently passed through a cannula (27 G) four times. After centrifugation for 20 min at 726 × *g* the supernatant was transferred into ultracentrifugation tubes. Ultracentrifugation was carried out in a L-60 ultracentrifuge at 110 561 × *g* for 30 min. The pellet was dissolved in 150–200 μl lysis buffer, and 50 μl aliquots were shock frozen in liquid nitrogen and stored at -80°C. Total cell lysates of transfected tsA-201 cells were prepared by adding 150–200 μl ice-cold cell lysis buffer (50 mM Tris⋅HCl pH 7.4, 150 mM NaCl, 1 mM EDTA, 1 % (v/v) Triton X-100, supplemented with protease inhibitors as above) to collected cells and slow rotation at 4°C for 20 min. Insoluble cell debris was removed by centrifugation for 15 min (16 600 × *g*) at 4°C. Aliquots of the lysate were shock frozen in liquid nitrogen and stored at -80°C.

Membrane protein preparation from adult mouse brain was performed as described (Pichler et al., 1997).

### SDS-PAGE and Western Blotting

Protein in sample buffer was denatured under reducing conditions at 57°C for 15 min. Samples and prestained molecular weight marker (Precision Plus Protein All Blue Standards, Biorad) were separated on polyacryamide gels (5, 12% gels; or 4-15% gradient gels) in 25 mM Tris Base, 192 mM glycine, 0.1% SDS. Separated proteins were blotted on polyvinylidene fluoride (PVDF) membrane [Immobilon-P Transfer membrane, Millipore; transfer buffer: 25 mM Tris base, 192 mM glycine, 20% (v/v) methanol, with or without 0.1% (w/v) SDS]. Coomassie staining of gels was performed to check for efficiency of the transfer. Immunostained bands were visualized using Pierce ECL Western Blotting Substrate (Thermo Scientific) and a Fusion Fx7 Peqlab bioimager. Quantitation of band intensity was performed with Image J 1.46 (National Institute of Health). For quantification integrated density of specific bands was normalized against loading control. Unspecific bands and Coomassie-stained membranes were used as loading control. Quantification of gel or blot intensities was performed with data obtained within a linear range of exposure.

### Immunohistochemistry

Cochleae of hearing Cav1.3DCRD^HA/HA^ mice and WT littermates (aged 3–11 weeks) were fixed by injection of Zamboni’s fixative into the round and oval window and incubation for 8 min on ice, followed by rinsing with PBS. The organ of Corti was dissected and mounted on a slide using CellTak (BD Bioscience). Whole-mounts were stained using the following solutions: PBS, blocking buffer (1% BSA in PBS), permeabilization buffer (0.5% Triton X-100 in PBS), reaction buffer (0.5% BSA, 0.2% Triton X-100 in PBS), washing buffer (0.1% Triton X-100 in PBS). Whole-mounts were embedded with Vectashield mounting medium with DAPI (Vector UK) and viewed using a confocal Zeiss LSM 700. Whole-mounts were double-labeled by simultaneous incubation of an Alexa488-conjugated anti-HA antibody and antibodies directed against Cav1.3, CtBP2/RIBEYE or Cavβ2 (see above), which were detected using a Cy3-conjugated secondary antibody (Jackson Immunoresearch).

### Electrophysiological Recordings in tsA-201 Cells

Cell culture, transfection and electrophysiological recordings were performed as described previously using 15 mM Ca^2+^ as charge carrier (Singh et al., 2008; Lieb et al., 2014). Recording solutions [in mM]: extracellular (bath) solution: 15 CaCl_2_, 10 HEPES, 150 choline-Cl, 1 MgCl_2_, adjusted to pH 7.4 with CsOH and intracellular (pipette) solution: 135 CsCl, 10 Cs-EGTA, 1 MgCl_2_ adjusted to pH 7.4 with CsOH. The voltage-dependence of activation was determined from current–voltage (I–V) – relationships obtained by step depolarizations from a holding potential of -80 mV to various test potentials. Data were fitted to the equation:

$$I=G_{\max}\left(V-V_{rev}\right)/(1+\exp\left[(V_{0.5}-V)/K_{act}\right]),$$

where V_rev_ is the extrapolated reversal potential of I_Ca_, V is the membrane potential, I is the peak current, G_max_ is the maximum conductance of the cell, V_0.5_ is the voltage for half maximal activation and k_act_ is the slope factor of the Boltzmann term. Data were corrected for the liquid junction potential (8.5 mV). The time course of Ca^2+^ current inactivation (I_Ca_) was assessed during a 5-s depolarizing testpulse to the voltage of maximal current influx (V_max_). The percentage of I_Ca_ inactivation was calculated at various time points (30 and 250 ms, 1 and 5 s).

### Electrophysiological Recordings in IHCs

Recordings were performed on mature apical turn IHCs from P18–P21 Cav1.3DCRD^HA/HA^ mice and their WT littermate controls. Animal procedures were approved by the Saarland University. Animals were killed by decapitation in accordance with national ethical guidelines. Organs of Corti were dissected and kept in extracellular solution with reduced Cl^-^ concentration containing (in mM): 70 NaOH⋅lactobionate, 83 NaCl, 5.8 KCl, 1.3 CaCl_2_, 0.95 MgCl_2_, 5.3 glucose, 10 HEPES, 0.7 NaH_2_PO_4_ (pH = 7.35; 320 mosmol kg^-1^). Ca^2+^ currents were recorded during superfusion with (in mM): 10 CaCl_2_, 35 tetraethylammonium (TEA) chloride, 15 4-aminopyridine (4-AP) and 100 μM linopirdine in order to block K^+^ currents. Ba^2+^ currents were recorded in extracellular solution containing (in mM): 72.5 NaOH⋅lactobionate, 40 NaCl, 0.9 MgCl_2_, 5.6 glucose, 10 HEPES, 10 BaCl_2_, 35 TEA, 15 4-AP (pH = 7.35; 320 mosmol kg^-1^). For all experiments quartz pipettes were used filled with (mM): 110 Cs^+^ methane sulfonate, 20 CsCl, 10 Na^+^ phosphocreatine, 5 HEPES, 5 EGTA, 4 MgCl_2_, 4 Na_2_ATP, 0.1 CaCl_2_, 0.3 GTP (pH 7.35, 305 mosmol kg^-1^). Whole-cell patch clamp recordings were performed at room temperature using an Optopatch (Cairn Research, Faversham, UK) and an AXOPATCH 200B amplifier (Molecular Devices, Palo Alto, CA, USA). Ca^2+^ and Ba^2+^ currents were corrected oﬄine for linear leak currents and for potentials by subtracting their respective liquid junction potential (6 mV for Ca^2+^ currents; 8 mV for Ba^2+^ currents; for data acquisitions see also Michna et al., 2003). Ca^2+^ (Ba^2+^) currents were elicited by 8 ms long depolarizing voltage steps from -99 mV to +46 mV (-97 mV to +48 mV) with 5 mV increments. *I-V* relations were calculated as the average current taken from the last ms of the voltage steps as a function of the respective voltage. Fits of *I–V* curves were performed as described (Pirone et al., 2014), yielding the voltage of half-maximum activation (V_0.5_) und the slope (k_act_) as a measure of voltage-sensitivity. Inactivation was measured by applying 400 ms long depolarizing steps to the voltages stated above with 10 mV increments. Currents at the voltage of maximum activation were fitted with a double-exponential function (Ca^2+^ currents) or a mono-exponential function (Ba^2+^ currents), respectively (Michna et al., 2003). For normalization, respective current values were divided by the peak current value at the starting point of the fit.

### Electrophysiological Recordings in Chromaffin Cells

Isolation and culture of chromaffin cells was performed as described (Marcantoni et al., 2010). Currents were recorded in perforated-patch conditions (Cesetti et al., 2003) using a multiclamp 700-B amplifier and pClamp 10.0 software (Molecular Devices, Sunnyvale, CA, USA). Traces were filtered using a low-pass bessel filter set at 1–2 kHz and sampled at 10 kHz using a digidata 1440 A acquisition interface (Molecular Devices, Sunnyvale, CA, USA). Borosillicate glass pipettes (Kimble Chase Life Science, Vineland, NJ, USA) with a resistance of 2–3 MΩ were dipped in an Eppendorf tube containing intracellular solution before being back-filled with the same solution containing 500 μg/ml of amphotericin B (Sigma Aldrich, Munich, Germany), dissolved in DMSO (Sigma Aldrich, Munich, Germany). Recordings were initiated after amphotericin B brought the access resistance below 15 MΩ (5–10 min) (Cesetti et al., 2003). Series resistance was compensated by 80% and monitored throughout the experiment. Fast capacitive transients during step-wise depolarisations (in voltage-clamp) were minimized online by the use of the patch-clamp analog compensation. Uncompensated capacitive currents (in voltage-clamp) were further reduced by the subtraction of the averaged currents in response to P/4 hyperpolarising pulses. Intracellular solution used for Ca^2+^ and Ba^2+^ current measurements was composed of (in mM) 135 Cs-MeSO_3_, 8 NaCl, 2 MgCl_2_ and 20 HEPES, pH 7,4 (with CsOH). The extracellular solution used was composed of (in mM) 135 TEA-Cl, 2 CaCl_2_ or 2 BaCl_2_, 2 MgCl_2_, 10 HEPES, 10 glucose, pH 7.4 (with TEA-OH). TTX (300 nM; Tocris Bioscience: Bristol, UK)) was added to avoid contamination by Na^+^ currents. L-type currents were obtained by subtracting the nifedipine (3 μM) - resistant component from total Ca^2+^ currents (Marcantoni et al., 2010). Solutions were perfused using a gravity based perfusion system. For current-clamp recording external solution consisted of (in mM): 130 NaCl, 4 KCl, 2 CaCl_2_, 2 MgCl_2_, 10 HEPES and 10 glucose; pH 7.4 (with NaOH). The intracellular solution consisted of (in mM) 135 KAsp, 8 NaCl, 20 HEPES, 2 MgCl, 5 EGTA, pH 7.4 (with NaOH).

### Brain Slice Electrophysiology

Brains from neonatal mice (P10–P15) were extracted shortly after decapitation. They were acutely sliced with a vibratome while submerged in highly oxygenated and chilled artificial cerebrospinal fluid (Dragicevic et al., 2014). Slices (250 μm) were stored in oxygenated ACSF at room temperature. Electrophysiological measurements were carried at 36°C in cell-attached configuration. Patch pipettes had a resistance of 6–8 MΩ and contained (in mM): 132 K-gluconate, 1 EGTA, 2 MgCl_2_, 2 NaCl, 10 HEPES, 2 MgATP, 2 NaGTP and 1 mg/ml biocytin. The pH of 7.2 was balanced with tetraethylammonium hydroxide. ACSF contained (in mM): 125 NaCl, 26 NaHCO_3_, 2.5 KCl, 1.25 NaHPO_4_, 2 CaCl_2_, 2 MgCl_2_. The pH of 7.4 was reached by saturating the ACSF with carbogen. The glucose concentration in ACSF was 25 mM for preparation and storage and 10 mM during the electrophysiological recordings. To block fast synaptic transmission, 10 μM DNQX (6,7 dinitroquinoxaline-2,3-dione, Tocris) and 10 μM gabazine (SR95531 hydrobromide, Tocris) were added to artificial CSF.

### Hearing Measurements

Auditory brainstem responses (ABR) and distortion product otoacoustic emissions (DPOAE) were recorded in anesthetized mice aged 3–5 weeks as described (Engel et al., 2006; Ruttiger et al., 2013). For anesthesia a mixture of ketamine-hydrochloride (75 mg/kg body weight, Ketavet 100, Pharmacia, Karlsruhe, Germany) and xylazine-hydrochloride (5 mg/kg body weight, Rompun 290, Bayer, Leverkusen, Germany) was injected intraperitoneally with an injection volume of 5 ml/kg b.w. Anesthesia was maintained by subcutaneous application of 1/3 of the initial dose, typically in 30 min intervals. Body temperature was maintained with a temperature-controlled heating pad. ABR thresholds were determined with click (100 μs) or pure tone stimuli (3 ms + 1 ms rise/fall time, 2–45 kHz) with electrodes placed at the ear (positive) and vertex. Cubic 2^∗^f1-f2 DPOAE amplitudes for the two stimulus primaries with frequencies f1 and f2 and f2 = 1.2^∗^f1, and sound pressure level L1 = 55 dB SPL and L2 = 45 dB SPL for the first and the second primary, respectively, were measured in the range between 10 and 18 kHz using 0.5 kHz steps followed by averaging (Schimmang et al., 2003; Hecker et al., 2011).

### Homecage Activity

Homecage activity was quantified using an automated system (Inframot; TSE, Bad Homburg, Germany) over a period of two light cycles and three dark cycles as previously described (Singewald et al., 2004; Cole et al., 2008)^.^ Measurement was started at the beginning of the dark cycle after 12 h of habituation. Eight animals were tracked simultaneously, each in a type 3 Makrolon cage (265 × 150 × 420 mm) by sensing the body heat image (infra-red radiation) and its spatial displacement over time. No movements were monitored when the mice were inactive, sleeping or during moderate self-grooming. Data were collected in bins of 1 min and were subsequently pooled to 1-h intervals.

### Statistics

Data analysis was performed using Clampfit 10.2 (Axon Instruments), Sigma Plot 11 (Systat Software Inc.), Statistica 8.0, Origin 6.0 or Igor Pro 6.12. All values are presented as mean ± SE for the indicated number of experiments (n), except if stated otherwise. Data were analyzed by unpaired Student’s *t*-test (Welch’s test for differing variances), Mann–Whitney test, or one-way ANOVA followed by Bonferroni *post hoc* test as indicated using Graph Pad Prism 5.1 software (GraphPad Software Inc.).

## Results

### HA-Tag in DCRD Disrupts Functional CTM in a Recombinant Cav1.3 Channel Construct

**FIGURE 1:**
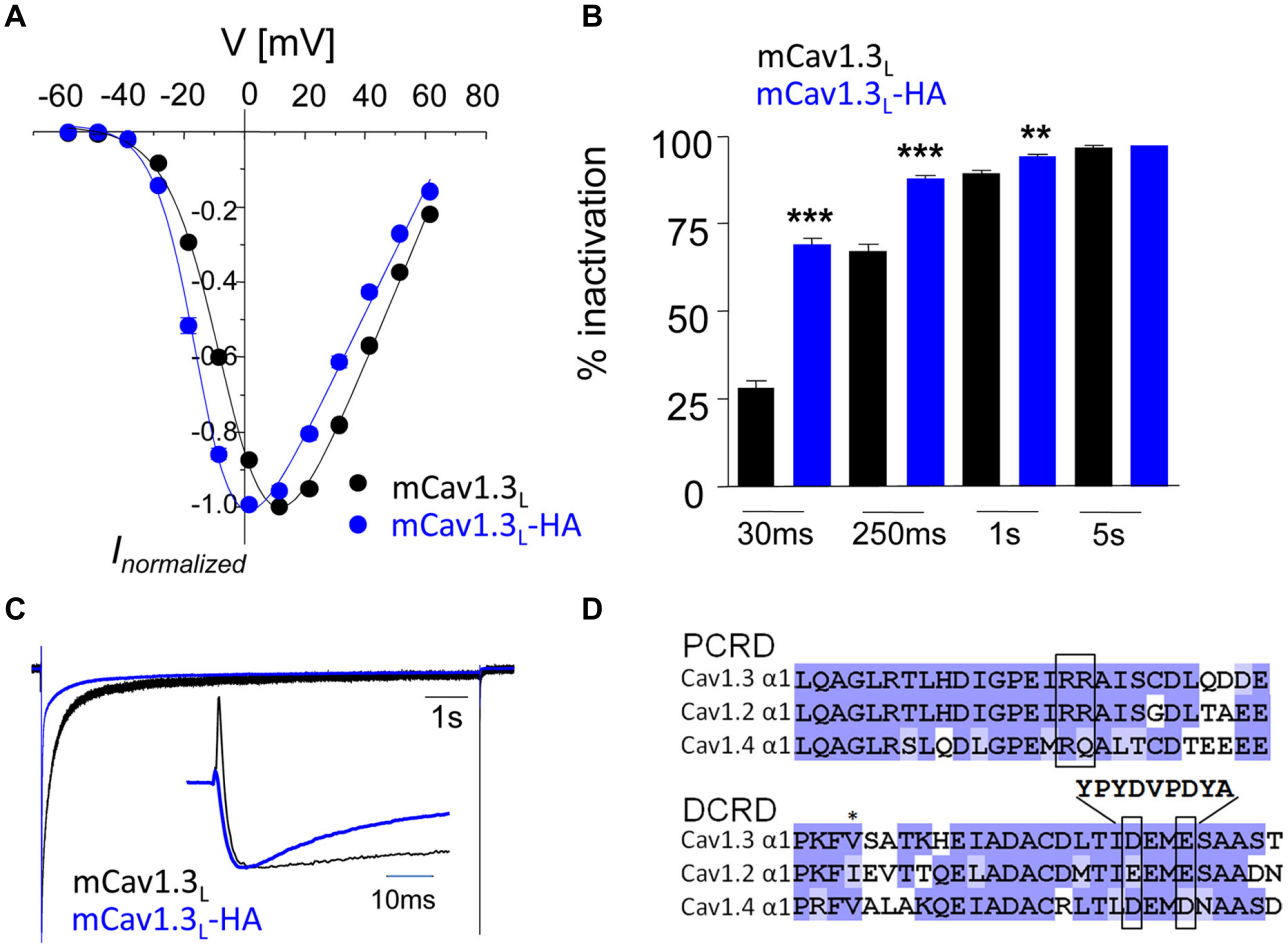
**Activation and inactivation properties of I_Ca_ through mCav1.3_L_ and mCav1.3_L_-HA channels. (A)** α1-subunits were heterologously expressed in tsA-201 cells together with β_3_ and α_2_δ_1_ (at least three independent transfections). Whole-cell patch-clamp current–voltage relationship obtained by depolarizations from a Vh of -80 mV to the indicated test potentials in cells transfected with mouse wild-type (WT) Cav1.3 (mCav1.3_L_, black) and mCav1.3_L_-HA (blue). All data were junction potential – corrected. **(B)** Percent I_Ca_ inactivation (15 mM Ca^2+^) during a test pulse from -80 mV to the V_max_. ^∗∗∗^*p* < 0.001; ^∗∗^*p* < 0.01 (one-way ANOVA analysis followed by Bonferroni post-test). Data are means ± SE (error bars often smaller than symbols). For gating parameters, *n*-numbers and statistics see **Table 1**. **(C)** Normalized I_Ca_ recordings in tsA-201 cells expressing mCav1.3_L_ or mCav1.3_L_-HA channel complexes. Cells were depolarized for 10 s from -90 mV to V_max_. The inset shows the first 50 ms of the test pulse. Notice the transient at the beginning of the pulse reflecting ON gating currents which were prominent in mCav1.3_L_ but absent or barely visible in Cav1.3DCRD^HA^ (indicating higher open probability of short Cav1.3 α1-isoforms in agreement with our previous findings; Bock et al., 2011; Lieb et al., 2014). Cells 1005090023, 1804090028. **(D)** To disrupt CTM function residues DEME (amino acids 2073-2076; NCBI accession number EU363339) were replaced by a single 9-residue HA-tag. Successful functional disruption was verified in electrophysiological experiments **(A–C)**. This can be explained by removal of one of the negative charges required for interaction with the PCRD by the HA-tag and as well as disruption of the putative α-helical structure in this region (as predicted using secondary structure prediction by Jpred; Cole et al., 2008).

We first inserted a hemagglutinin (HA)-tag into the recombinant mouse Cav1.3 α1-subunit (long splice variant, mCav1.3_L_) and verified that this strategy disrupts CTM modulation in transfected tsA-201 cells (**Figure 1**). In this construct (mCav1.3_L_-HA) we replaced critical negative charges within the DCRD region (amino acids DEME, Singh et al., 2008; see Materials and Methods and **Figure 1D**) with a sequence encoding the HA-epitope without truncating the C-terminus. mCav1.3_L_-HA fully reproduced the gating behavior of short Cav1.3 splice variants (Bock et al., 2011). Compared to WT Cav1.3 Ca^2+^ currents (I_Ca_), mCav1.3_L_-HA currents exhibited significantly stronger voltage-dependence of activation (**Figure 1A**), faster inactivation (**Figures 1B,C**) and higher current densities (**Table 1**). As in short Cav1.3 splice variants, mCav1.3_L_-HA ON-gating currents were absent or only small despite robust inward I_Ca_, due to the higher open probability of short Cav1.3 channels (**Figure 1C**; Bock et al., 2011; Lieb et al., 2014). These data demonstrate that the HA-insertion in the DCRD successfully blocked CTM function and conferred the biochemically long Cav1.3 channel isoform with gating properties expected for short isoforms. Introduction of the HA-tag did not interfere with efficient α1-subunit expression as a full- length protein in tsA-201 cells (221 kDa ± 7 kDa, SD, *n* = 3). In the mutant C-terminus still all other functional domains, including a PDZ-binding motif at the C-terminal end (Jenkins et al., 2010) are preserved. Since the genetic modification affects the last exon and the remaining loxP site is located in the 3′-UTR alternative splicing of the channel should not be affected. Mice containing this modification should therefore only report changes resulting from altered channel gating induced by CTM disruption.

**Table 1:** Biophysical properties of mCav1.3_L_ and mCav1.3_L_-HA.

	Parameter	mCav1.3_L_			mCav1.3_L_-HA			*p*
			
		Mean	±SD	*n*	Mean	±SD	*n*	
I_Ca_	I_max_ [pA/pF]	50.22	±36.72	36	86.94	±33.40	8	<0.01
	V_0.5_ [mV]	-4.09	±2.47	36	-13.76	±2.00	8	<0.0001
	k_act_ [mV]	8.87	±0.66	36	6.94	±0.81	8	<0.0001
	Inactivation [%]	66.26	±11.17	30	88.16	±1.95	7	<0.0001
	τ_fast_ [ms]	14.92	±3.65	30	12.63	±1.98	7	0.0156
	τ_slow_ [ms]	207.23	±77.25	30	117.39	±16.27	7	<0.0001
	A_fast_	0.23	±0.12	30	0.75	±0.02	7	<0.0001
	A_slow_	0.64	±0.11	30	0.15	±0.01	7	<0.0001
	Non-inact	0.13	±0.04	30	0.10	±0.01	7	<0.0001


*Cav1.3 channels were expressed in tsA-201 cells and activation parameters were obtained from I–V curves fitted as described in methods. V_0.5_, voltage of half-maximum activation; k_act_, steepness of activation curve; Inactivation %, percent inactivation 250 ms after time of peak current. Non-linear curve fits to a double-exponential function revealed time constants for fast (τ_fast_) and slow (τ_slow_) inactivation during 300 ms, and the fraction of I_Ca_ normalized to I_max_ with fast (A_fast_) and slow (A_slow_) inactivation and of a non-inactivating component (non-inact). Data were fitted to a double-exponential function. Welch’s t-test was performed for statistical analysis.*

### Generation of Cav1.3DCRD^HA/HA^ Mice

**FIGURE 2:**
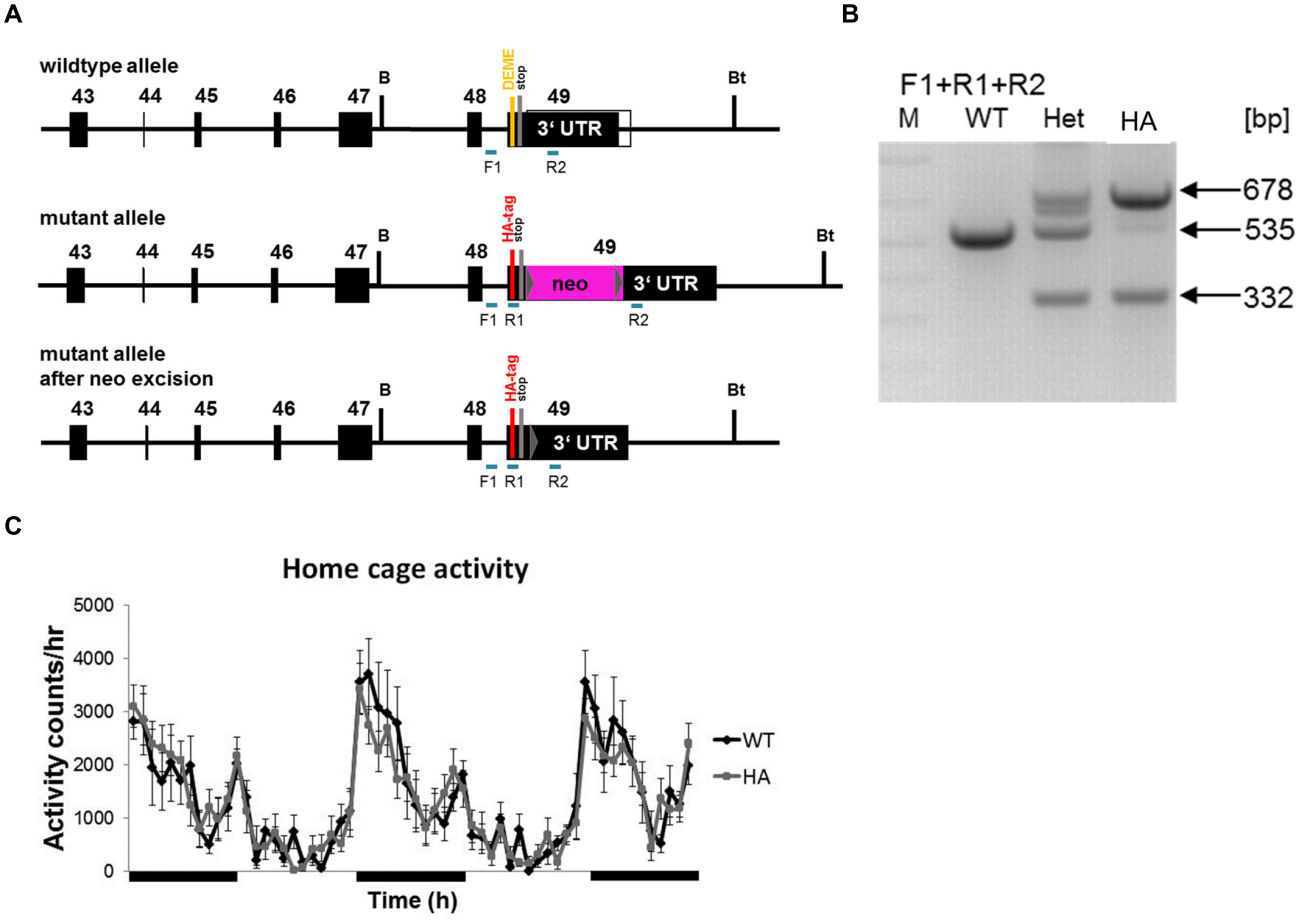
**Gene targeting strategy to generate Cav1.3DCRD^HA/HA^ mice. (A)** PCR primer positions for genotyping (see Materials and Methods) are indicated. WT allele, mutated allele and mutated allele after neo excision are shown. DEME, single amino acid letter code for negatively charged DCRD sequence replaced by HA-tag as indicated. F1, R1, R2 indicate forward and reverse primers used for genotyping. **(B)** Genotyping reactions yielding the expected fragment sizes are shown for WT, hetero- (Het) and homozygous (HA) mutant mice. **(C)** Hourly time course of home cage activity in HA (*n* = 10, gray) and WT mice (*n* = 11, black) recorded over three consecutive dark and two light cycles as described in Methods. Black bars indicate dark periods. Data are presented as Mean ± SE.

We introduced the identical modification of the DCRD region in exon 49 of the murine *cacna1d* gene (**Figure 2**). The resulting homozygous mutants (Cav1.3DCRD^HA/HA^ mice; *neo*-cassette removed) were viable and showed normal sexual activity and reproduction. No gross anatomical or behavioral abnormalities were observed. Litters from heterozygous mice showed normal Mendelian inheritance. The mutation did not affect spontaneous locomotor activity (homecage activity, **Figure 2C**).

### Short and Long Cav1.3 α1-Variants are Expressed in Mouse Brain

**FIGURE 3:**
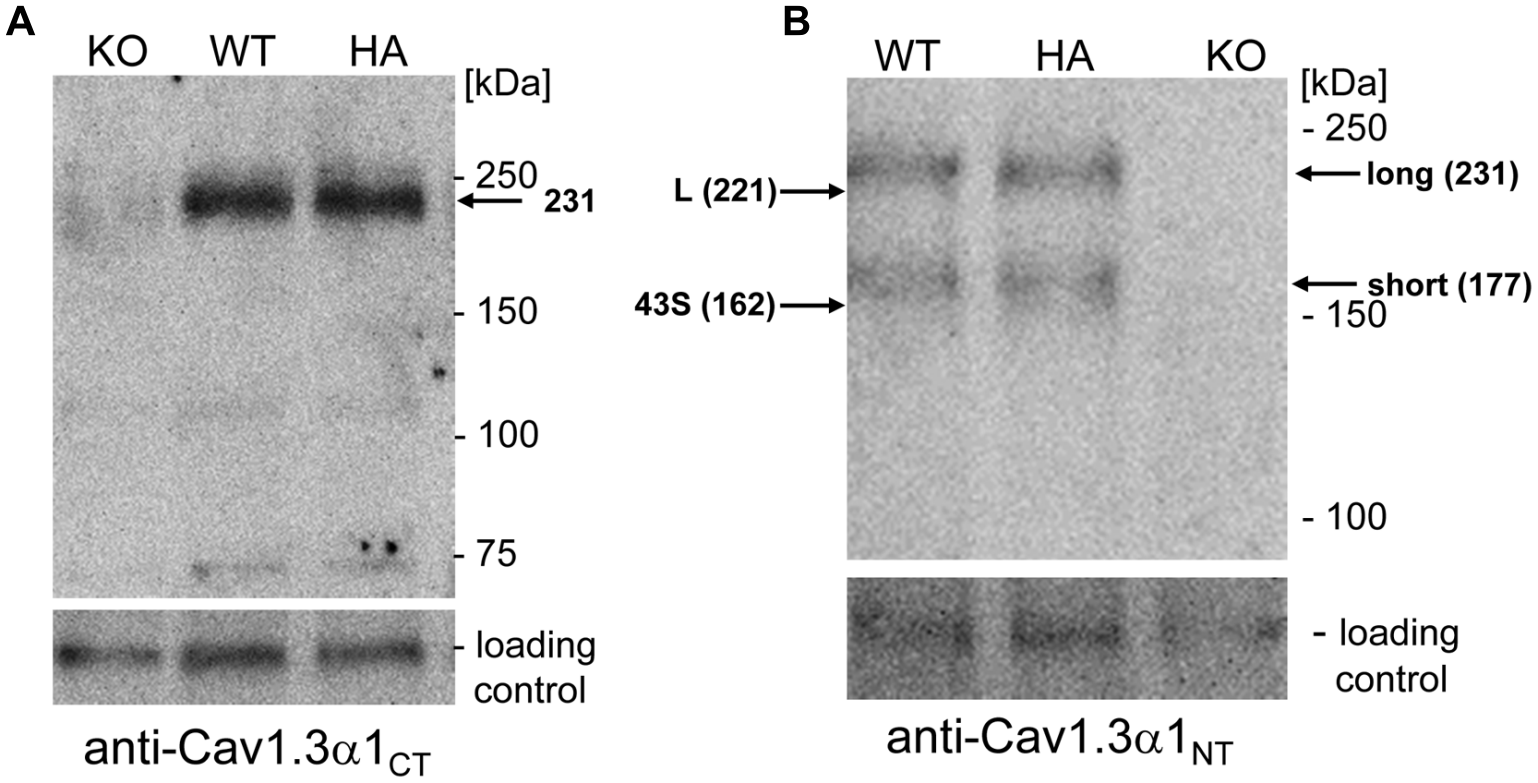
**Western blot analysis of α1-subunits in WT and mutant mouse brain homogenates. (A)** Proteins (100 μg/lane) were separated on 5% SDS page and immunostained with polyclonal antibody Cav1.3 α1_CT_. The Cav1.3 α1 subunit was specifically detected as a 231 kDa protein at expression levels indistinguishable between WT and homozygous mutants (HA) (see Results). KO, Cav1.3^-/-^ negative control. **(B)** Same separation as in A (100 μg/lane) but detection with anti-Cav1.3α1_NT_. The migration of recombinant mCav1.3_L_ (L) and mCav1.343_S_ (43S) on the same gel (not shown) and their calculated molecular mass are indicated by arrows (left). Migration of molecular mass standards as well as the brain long and short α1-subunit species are also indicated (right). An unspecific ∼120 kDa band served as loading control. One representative experiment of at least three independent experiments is shown for all panels. KO, Cav1.3^-/-^; HA, Cav1.3DCRD^HA/HA^, WT, wild-type littermate.

First we confirmed that the HA-tagged Cav1.3 α1-subunit is expressed as a full-length protein *in vivo* and that the HA-tag did not alter the level of overall Cav1.3 α1 protein expression. Western blot analysis of whole brain homogenates (*n* > 3, **Figure 3**) immunostained with a C-terminal antibody (anti-Cav1.3α1_CT_; Platzer et al., 2000) revealed equal Cav1.3 α1-subunit expression levels in homozygous mutants (Cav1.3DCRD^HA/HA^: 111% ± 19%; mean ± SD, *n* = 3) as compared to their WT littermates (**Figure 3A**). Specificity of the antibody was demonstrated in Cav1.3^-/-^ brains analyzed in parallel.

In previous reports two size forms of Cav1.3 α1-subunits were detected in rodent brain (Hell et al., 1993; Calin-Jageman et al., 2007). Since anti-Cav1.3α1_CT_ (directed against the distal C-terminus) and anti-HA antibodies only bind to the long Cav1.3 splice variant, an N-terminal antibody recognizing all Cav1.3 α1 subunits (anti-Cav1.3α1_NT_) in postnatal brain was employed to quantify the presence of shorter variants. In addition to the HA-tagged α1 (apparent mass 231 kDa, **Figure 3**) this antibody also specifically detected a shorter α1-subunit variant (177 kDa, *n* = 6) of equal staining intensity and no change in the ratio of the two size forms in Cav1.3DCRD^HA/HA^ mice (**Figure 3B**; long species: WT: 49 ± 8% of total immunoreactivity; Cav1.3DCRD^HA/HA^, 47 ± 5%; mean ± SD, *n* = 6). The absence of a smaller HA-stained species (**Figure 3A**) ruled out that short forms (detected with anti-Cav1.3α1_NT_ in **Figure 3B**) contain exon 49 sequence and must therefore correspond to C-terminally short variants lacking a DCRD domain. The two α1-species identified in brain migrated with slightly larger apparent molecular masses (231 and 177 kDa, **Figure 3B**) than the recombinant long (L) and short (43S, Cav1.3_43S_, Bock et al., 2011) α1-subunits separated on the same gel (221 and 162 kDa, L and 43S, arrows in **Figure 3B**). Because differences in glycosylation are unlikely (tsA-201 cells allow efficient glycosylation) we propose that the Cav1.3 α1-subunits in brain extensively utilize additive alternative splicing of exons not present in our recombinant constructs (such as exons 11, 32, and 44). Although absolute molecular masses are difficult to determine due to the abnormal migration of Ca^2+^ channel α1-subunits in SDS-PAGE (Glossmann et al., 1988), the difference between the long and short brain α1-subunit bands (brain: 54 kDa) is close to the calculated (53 kDa; Cav1.3_L_ 244 kDa; Cav1.3_43S_ 191 kDa; Bock et al., 2011) and measured (tsA-201 cells: 59 kDa) molecular mass difference between Cav1.3_L_ and Cav1.3_43S_.

### No Evidence for a Stable C-Terminal Proteolytic Fragment in Mouse Brain

**FIGURE 4:**
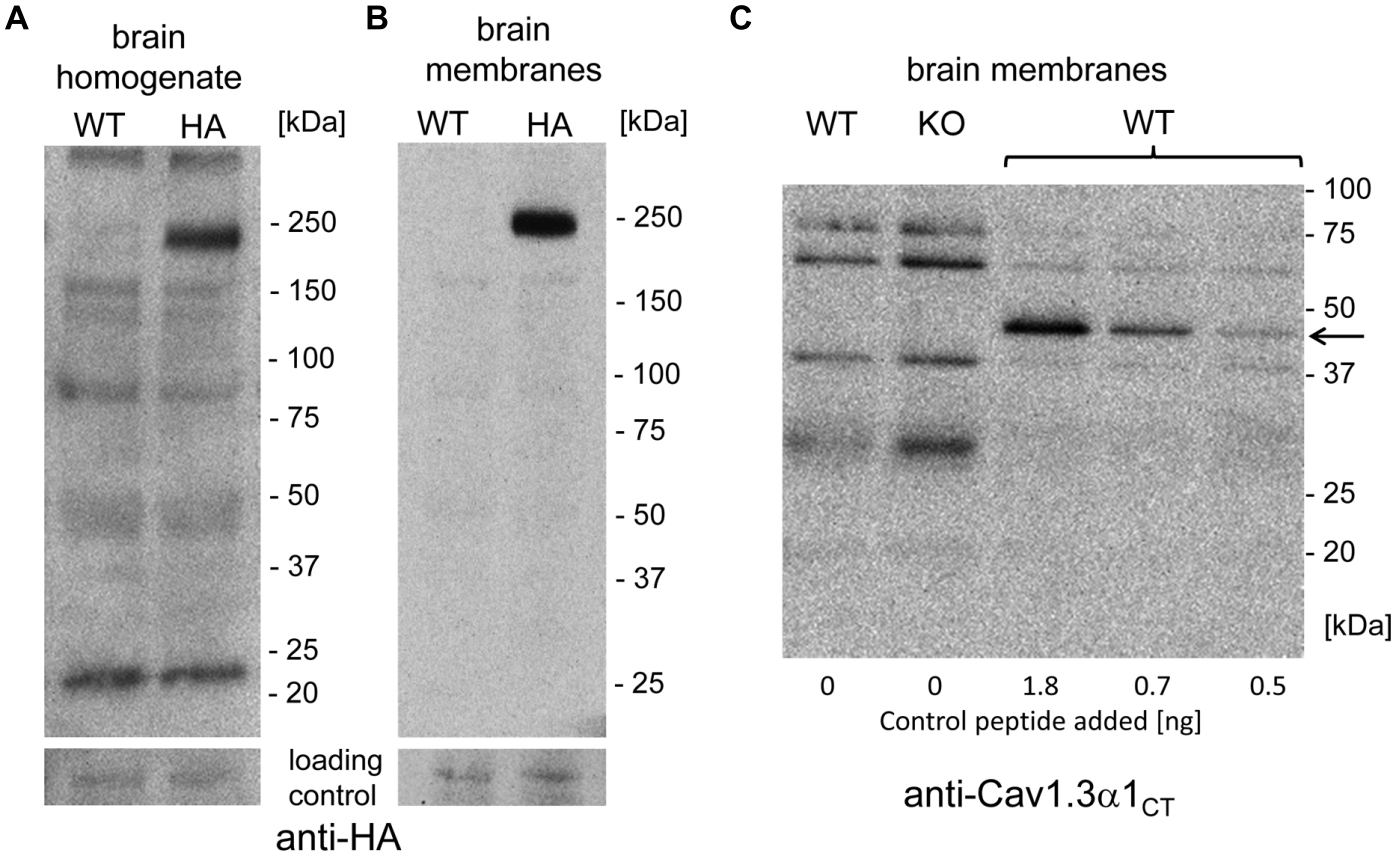
**Absence of smaller C-terminally–derived Cav1.3 α1 fragments in WT and Cav1.3DCRD^HA/HA^ brain preparations. (A)** Mouse brain homogenate (100 μg of protein/lane) prepared from WT or Cav1.3DCRD^HA/HA^ (HA) mice were separated on 4–15% gradient SDS-PAGE and immunostained with anti-HA antibody. The blot was overexposed to also visualize less abundant smaller fragments. in separate experiments α1- associated HA-immunoreactivity could be detected with only 10% (10 μg/lane) of the protein amount used (*n* = 3) demonstrating the sensitivity of the assay. **(B)** Mouse brain membranes (100 μg of protein/lane) were analyzed as in **(A). (C)** Mouse brain membranes (100 μg of protein/lane) from WT or Cav1.3^-/-^ (KO) mice were blotted as in **(B)** and stained with anti-Cav1.3α1_CT_ antibodies. To some WT samples (33 μg/lane) a 45 kDa recombinant C-terminal control peptide was added (arrow, amounts indicated) before separation to demonstrate successful transfer and sensitive detection as a positive control for sensitivity.

The short Cav1.3 α1-subunit species could arise either from alternative splicing or from C-terminal post-translational proteolytic processing thereby generating stable C-terminal peptides as demonstrated for Ca_V_1.1 and Cav1.2 α1-subunits (Hulme et al., 2005, 2006; Gomez-Ospina et al., 2006). If this was also the case for Cav1.3 α1 in brain, then one or more smaller HA-tagged C-terminal peptides should be detectable in mutant mice. Cleavage at the proposed site of Cav1.1 and Cav1.2 α1 subunit (amino acid 1800, NCBI reference NP_001242928.1; Hulme et al., 2005, 2006), well conserved in Cav1.3, would lead to a 399 amino acid peptide (45 kDa), whereas a fragment accounting for the mass difference of the two size forms would lead to a 53 kDa peptide. Neither anti-HA (brain homogenate or brain membranes; comparison against WT; **Figures 4A,B**) nor anti-Cav1.3α1_CT_ antibodies (comparison against knockout control, **Figure 4C**) specifically detected smaller candidate peptides (three independent experiments from three different brain preparations). However, in control experiments anti-Cav1.3α1_CT_ specifically recognized small amounts of a recombinant C-terminal Cav1.3 fragment (C-terminal 158 residues fused to GFP, 45 kDa, Singh et al., 2008) added to brain preparations before SDS-PAGE serving as a positive control for assay sensitivity (**Figure 4C**).

Taken together, we obtained no evidence for the presence of a stable HA-labeled C-terminal cleavage product. Instead, our experiments are in good agreement with our previous finding that about half of the Cav1.3 α1-subunit transcripts in brain encode short splice variants of almost identical size (mainly Cav1.3_42A_ and Cav1.3_43S_, Bock et al., 2011). Although contribution by proteolytic processing cannot be ruled out, our biochemical data using Cav1.3DCRD^HA/HA^ mice strongly indicate that the majority of short α1-subunit species is derived from alternative splicing.

### Role of Long Cav1.3 Channels for IHC Function and Hearing

**FIGURE 5:**
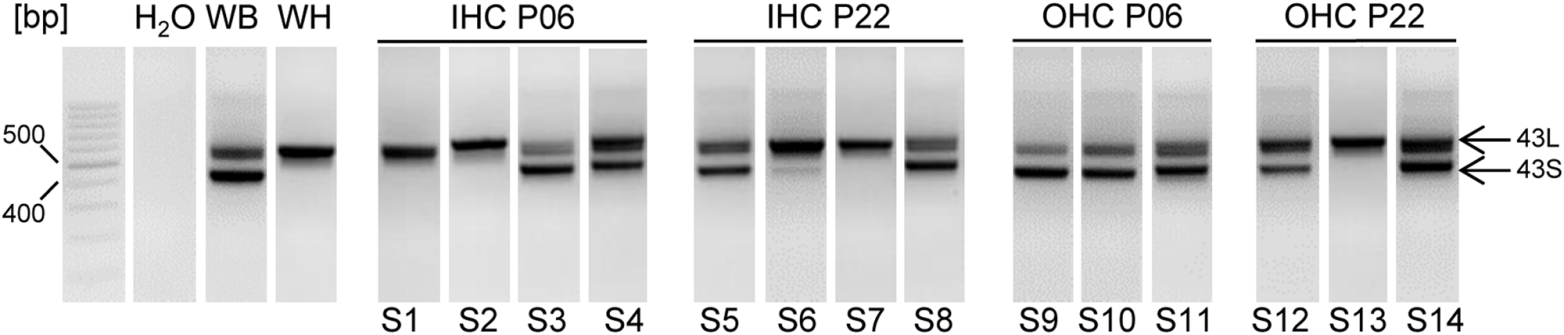
**Cav1.3 α1 transcripts containing exons 43_S_ and 43_L_ in mouse IHCs and OHCs, at P6 and P22 using nested PCR.** Fragments containing 43S (403 bp) or 43L (557 bp) were amplified using nested PCR (see Materials and Methods) with primers specific for exon 42 (forward) and 45 (reverse) of mouse Cav1.3. S1–S14 represent samples from independent preparations. For each cell type and developmental stage at least three independent experiments were performed. Whole brain (WB) and heart (WH) served as positive controls, H_2_O (no template) as negative control. Specificity of PCR products was confirmed by sequencing. When two independent PCR reactions with three different RNA samples of each cell type were performed, the number of successful detections for each transcript was as follows: detection of 43L: 6 (out of six experiments) in IHC and OHC preparations of all developmental stages; detection of 43S: 4 (6) in IHC P06 and IHC P22, 6 (6) in OHC P06 and 5 (6) in OHC P22. Bp, basepair markers.

To study the role of CTM function in intact cells we focused on IHCs and MCCs. In these cells the contribution of Cav1.3 current components to total I_Ca_ has been well defined (Platzer et al., 2000; Brandt et al., 2003; Marcantoni et al., 2010; Vandael et al., 2012). Moreover, the extent of CDI is very different in the two cell types because CaBPs strongly inhibit CDI in IHCs (Yang et al., 2006; Cui et al., 2007; Schrauwen et al., 2012) but not in chromaffin cells (Marcantoni et al., 2010). In addition, transcripts for both long and short Cav1.3 α1 splice variants are expressed in MCCs (Marcantoni et al., 2010) and, as shown in **Figure 5**, also in individual IHCs and OHCs.

**FIGURE 6:**
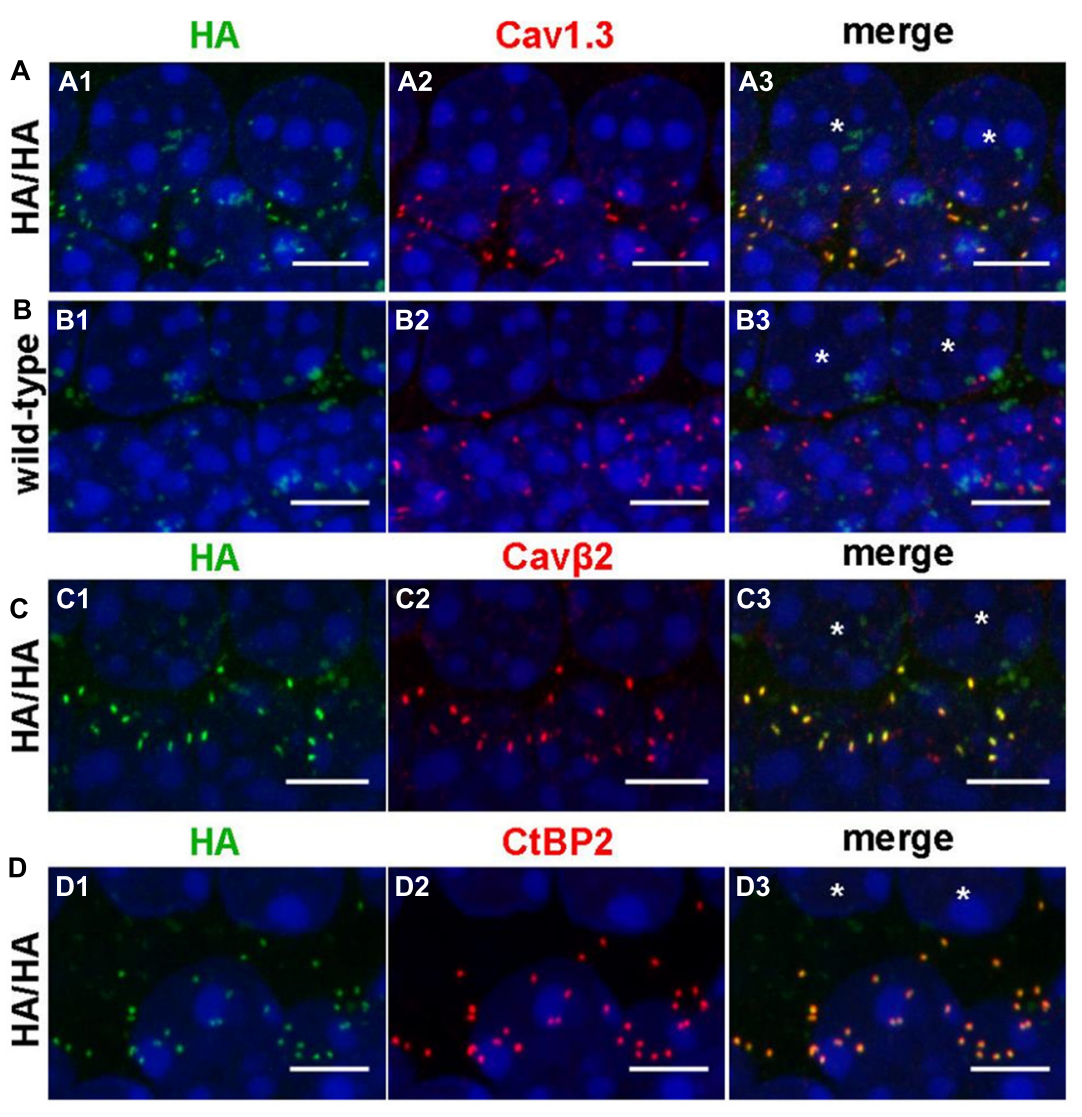
**Comparison of the protein localization of anti-HA-labeled Cav1.3 with immunolabeled Ca_v_1.3, Ca_v_β2 and CtBP2/RIBEYE in IHCs. (A–D)** Whole-mount preparations of apical turns of the organ of Corti from adult Ca_v_1.3DCRD^HA/HA^ **(A,C,D)** and WT **(B)** mice were co-immunolabeled with anti-HA and anti-Ca_v_1.3 (**A, B**, 11 weeks), anti-HA and anti-Ca_v_β2 (**C**, P28), or anti-HA and anti-CtBP2/RIBEYE antibodies (**D**, P37). Every image shows the basolateral poles of two adjacent IHCs the nuclei of which are indicated by asterisks in the rightmost column, respectively. HA staining **(A1,C1,D1)** largely overlapped with Ca_v_1.3 **(A2)**, Ca_v_ß2 **(C2)** and CtBP2 **(D2)** staining at the basal poles of IHCs as evident upon merging corresponding images **(A3,C3,D3)**. In the WT, no specific HA-labeling **(B1)** was present at the position of Ca_v_1.3 labeling **(B2,B3)**. The weak ‘cloudy’ green anti-HA staining was present in all specimen investigated and therefore considered unspecific. Cell nuclei of IHCs were counterstained with DAPI (blue). 1 of 3 (**A**, age: 2–3 months), 1 of 4 (**B**, age: P25 – 3 month), 1 of 5 (**C**, P25–P31) and 1 of 5 (**D**, P28–P37) independent experiments is illustrated, respectively. Scale bars: 5 μm.

We verified the presence of HA-tagged Cav1.3 channels in IHCs by whole-mount immunolabeling of adult Cav1.3DCRD^HA/HA^ organs of Corti with anti-HA antibodies using WT littermates as negative control (**Figure 6**). HA-labeled structures co-localized with immunolabeled Cav1.3 and Cavβ2, the main auxiliary β-subunit in IHCs (**Figures 6A,C**; Neef et al., 2009). In control experiments of WT specimens no comparable HA-immunoreactivity was observed demonstrating the specificity of the anti-HA antibody (**Figure 6B**). The anti-Cav1.3 antibody used (Alomone Labs, Israel) recognizes a stretch in the cytoplasmic II–III loop and thus detects the full length Cav1.3 channel (including HA-tagged channels) as well as C-terminally short isoforms (such as Cav1.3_43S_). The close corresponding staining of anti-Cav1.3 and anti-HA suggests that in adult IHCs all Cav1.3 clusters contain long Cav1.3 (**Figure 6A**). HA-tagged channels of mature IHCs were localized in very close apposition to synaptic ribbons as demonstrated by co-labeling with anti-CtBP2 (C-terminal binding protein 2), a specific marker for ribbon synapses (**Figure 6D**), indicating that long splice variants are present at all ribbon synapses.

**FIGURE 7:**
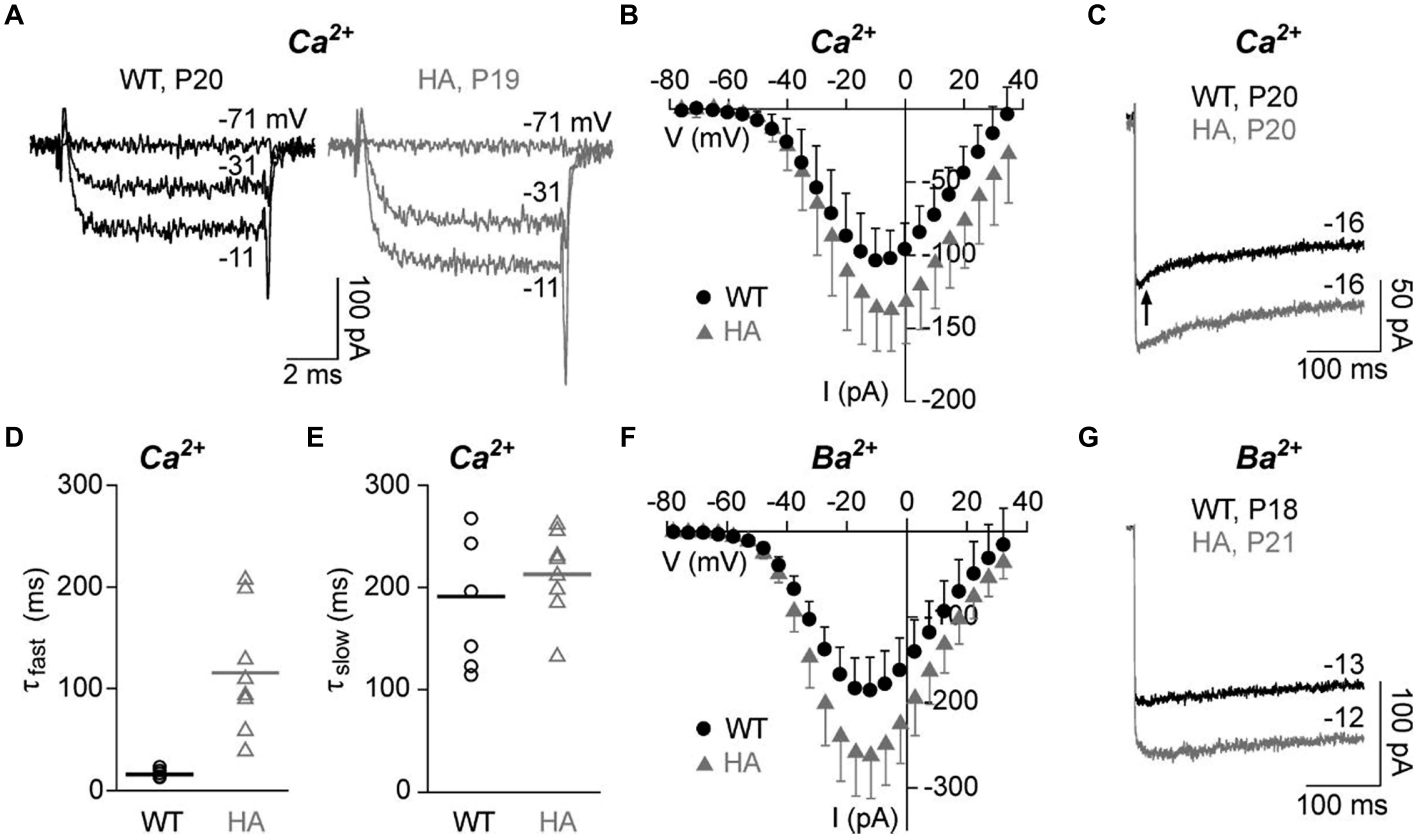
**Ca^2+^ and Ba^2+^ currents in adult IHCs (P18–21) of Cav1.3DCRD^HA/HA^ and WT littermates. (A)** Selected I_Ca_ traces (10 mM Ca^2+^) of a WT (black) and a Cav1.3DCRD^HA/HA^ (HA, gray) mouse IHC after depolarization from a holding potential of -86 mV for 8 ms to the indicated voltages. **(B)** Corresponding averaged I–V curves from 10 WT (black, mean + SD) and 12 Cav1.3DCRD^HA/HA^ IHCs (gray, mean – SD) taken between 7 and 8 ms after start of the depolarizing pulse. **(C)** Representative traces of I_Ca_ inactivation of WT (black) and a Cav1.3DCRD^HA/HA^ (gray) IHC during a 300-ms depolarizing step to V_max_. The arrow indicates the early phase with faster inactivation absent in Cav1.3DCRD^HA/HA^ and in Ba^2+^ recordings. **(D)** Parameters of I_Ca_ inactivation of 8 WT and 8 Cav1.3DCRD^HA/HA^ IHCs were obtained by fitting peak current traces from 300 – ms test pulses with a double-exponential function. Individual data points and means (lines) are shown. The fast (τ_fast_) inactivation time constant was significantly increased (*p* < 0.01) and showed a higher variance between individual IHCs in Cav1.3DCRD^HA/HA^ IHCs. **(E)** The slow (τ_slow_) inactivation time constant was not different between genotypes. **(F)** Averaged I–V curves of I_Ba_ with 10 mM Ba^2+^ as charge carrier from 9 WT (black, mean + SD) and 8 Cav1.3DCRD^HA/HA^ (gray, mean – SD) IHCs between 7 and 8 ms after start of the depolarizing pulse. **(G)** Representative traces of I_Ba_ inactivation of a WT (black) and a Cav1.3DCRD^HA/HA^ (gray) IHC during a 300 ms depolarizing step to V_max_.

To test if the disruption of CTM affects IHC Ca^2+^ currents, we performed patch-clamp recordings of mature IHCs with either 10 mM Ca^2+^ (I_Ca_, **Figures 7A–E**) or 10 mM Ba^2+^ (I_Ba_, **Figures 7F,G**) as charge carrier to also quantitate CDI. Depolarizations to the indicated voltages resulted in fast activating and deactivating inward currents for both genotypes. Peak I_Ca_ amplitudes and I_Ca_ current densities were significantly larger in Cav1.3DCRD^HA/HA^ IHCs as measured in averaged *I–V* relations (**Figure 7B**; **Table 2**). Membrane capacitance, a measure for IHC size, and parameters of I_Ca_ and I_Ba_ activation obtained from *I–V* relations (half-maximal activation voltage, slope of current activation) were not significantly altered (**Table 2**). During longer pulses (300 ms) WT I_Ca_ inactivated with a fast (τ_fast_, in the range of tens of ms) and a slow time constant (τ_slow_, in the range of hundreds of ms) (**Figures 7C–E**). In WT cells about 38% of the total current inactivated and τ_fast_ (A_fast_) contributed about 25% of the inactivation (corresponding to only about 10% of total; **Table 2**). In Cav1.3DCRD^HA/HA^ IHCs, τ_fast_ was significantly larger (**Figures 7C,D**; **Table 2**) and showed a larger variance than τ_slow_ suggesting that in these IHCs the fast inactivation process was strongly reduced but not completely abolished. τ_slow_ was not affected by the mutation (**Figure 7E**). Like I_Ca_, peak I_Ba_ was also significantly larger in Cav1.3DCRD^HA/HA^ IHCs (**Figure 7G**). Inactivation of I_Ba_, which mostly reflects VDI (Ben-Johny and Yue, 2014) was very slow during 300-ms depolarizations, decayed monoexponentially and did not differ between genotypes (**Figure 7G**; **Table 2**). Our data show that the small fast inactivating component in WT IHCs represents a component of CDI that, in contrast to predictions from studies with recombinant channels, is abolished when CTM function is disrupted in Cav1.3DCRD^HA/HA^ mice.

**Table 2:** Properties of IHC Ca^2+^ channel currents of Cav1.3DCRD^HA/HA^ mice and wild-type (WT) littermates.

	Parameter	WT			Cav1.3DCRD^HA/HA^			*p*
			
		Mean	± SD	*n*	Mean	±SD	*n*	
I_Ca_	C_m_ [pF]	9.85	±1.23	10	10.65	±1.77	11	0.105
	I_max_ [pA]	-102.08	±23.20	10	-135.95	±25.40	11	0.005
	Current density [pA/pF]	-10.54	±2.86	10	-13.06	±3.08	11	0.033
	V_0.5_ [mV]	-29.79	±3.09	10	-29.47	±2.70	11	0.801
	V_s_ [mV]	11.62	±1.15	10	11.23	±1.15	11	0.445
	Inactivation [%]	32.19	±13.33	8	26.34	±7.18	8	0.299
	τ_fast_ [ms]	16.06	±3.86	8	115.68	±60.59	8	0.002
	τ_slow_ [ms]	191.13	±58.26	8	212.97	±41.73	8	0.405
	A_fast_, _normalized_ [I/I_max_]	0.10	±0.04	8	0.14	±0.05	8	0.096
	A_slow_, _normalized_ [I/I_max_]	0.28	±0.10	8	0.17	±0.06	8	0.020
	Non-inact_normalized_ [I/I_max_]	0.62	±0.13	8	0.67	±0.09	8	0.347
I_Ba_	C_m_ [pF]	8.88	±1.74	9	10.25	±3.20	8	0.305
	I_max_ [pA]	-186.57	±37.49	9	-262.29	±48.82	8	0.003
	Current density [pA/pF]	-21.74	±5.73	9	-26.92	±7.03	8	0.076
	V_0.5act_ [mV]	-31.01	±2.28	9	-31.34	±1.67	8	0.737
	V_s_ [mV]	11.18	±0.89	9	10.72	±0.67	8	0.235
	Inactivation [%]	6.25	±2.60	8	6.91	±2.90	8	0.643
	τ [ms]	375.37	±98.37	8	410.27	±67.43	8	0.423
	A_normalized_ [I/I_max_]	0.12	±0.04	8	0.14	±0.04	8	0.379
	Non-inact_normalized_ [I/I_max_]	0.89	±0.04	8	0.87	±0.05	8	0.350

*C_m_, membrane capacitance; I_max_, current at voltage of maximum activation; V_0.5_, voltage of half-maximum activation; Inactivation %, percent inactivation 300 ms after time of peak current; all other parameters are as in **Table 1**. Student’s t-test or Mann-Whitney test were performed for statistical analysis.*

**FIGURE 8:**
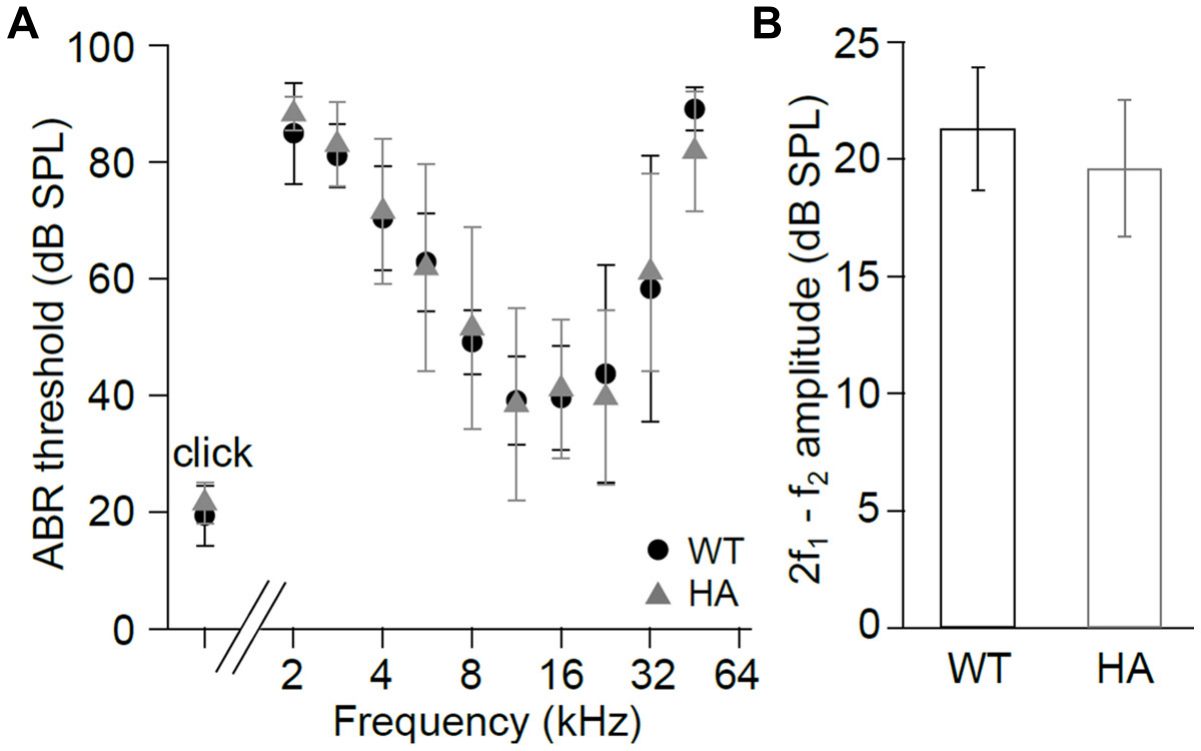
**Hearing function in Cav1.3DCRD^HA/HA^ mice. (A)** ABR thresholds (mean ± SD) of Cav1.3DCRD^HA/HA^ mice were normal for click stimuli (WT and Cav1.3DCRD^HA/HA^: *n* = 8/16 animals/ears) and as a function of stimulus tone frequency (WT: *n* = 6/12 animals/ears and Cav1.3DCRD^HA/HA^: *n* = 7/13 animals/ears). Thresholds could be measured in (*n* = animals/ears, frequency in kHz): WT: 2/3, 2; 6/9, 2.8; 6/12, from 4 to 22.6; 3/6, 32; 3/6, 45.2; Cav1.3DCRD^HA/HA^: 2/3, 2; 7/13, from 2.8 to 32; 4/8, 45.2. **(B)** Mean DPOAE maximum amplitudes (signal to noise ratio) ± SD at f1 = 9.1 kHz, L1 = 55 dB SPL, f2 averaged over 10–18 kHz, and L2 = 45 dB SPL were normal in Cav1.3DCRD^HA/HA^ (*n* = 7/14 animals/ears) compared with WT mice (*n* = 7/14 animals/ears, *p* = 0.12), indicating normal function of the cochlear amplifier.

To test if these changes in current properties also affect hearing function we recorded ABR (**Figure 8A**) and DPOAE (**Figure 8B**). Thresholds of click and frequency ABR recordings and DPOAE amplitudes were not changed in Cav1.3DCRD^HA/HA^ compared with WT mice. The data indicate that reduced CDI in Cav1.3DCRD^HA/HA^ IHCs does not cause detectable changes in hearing thresholds and the cochlear amplifier.

### Role of Long Cav1.3 Channels for Chromaffin Cell Function

**FIGURE 9:**
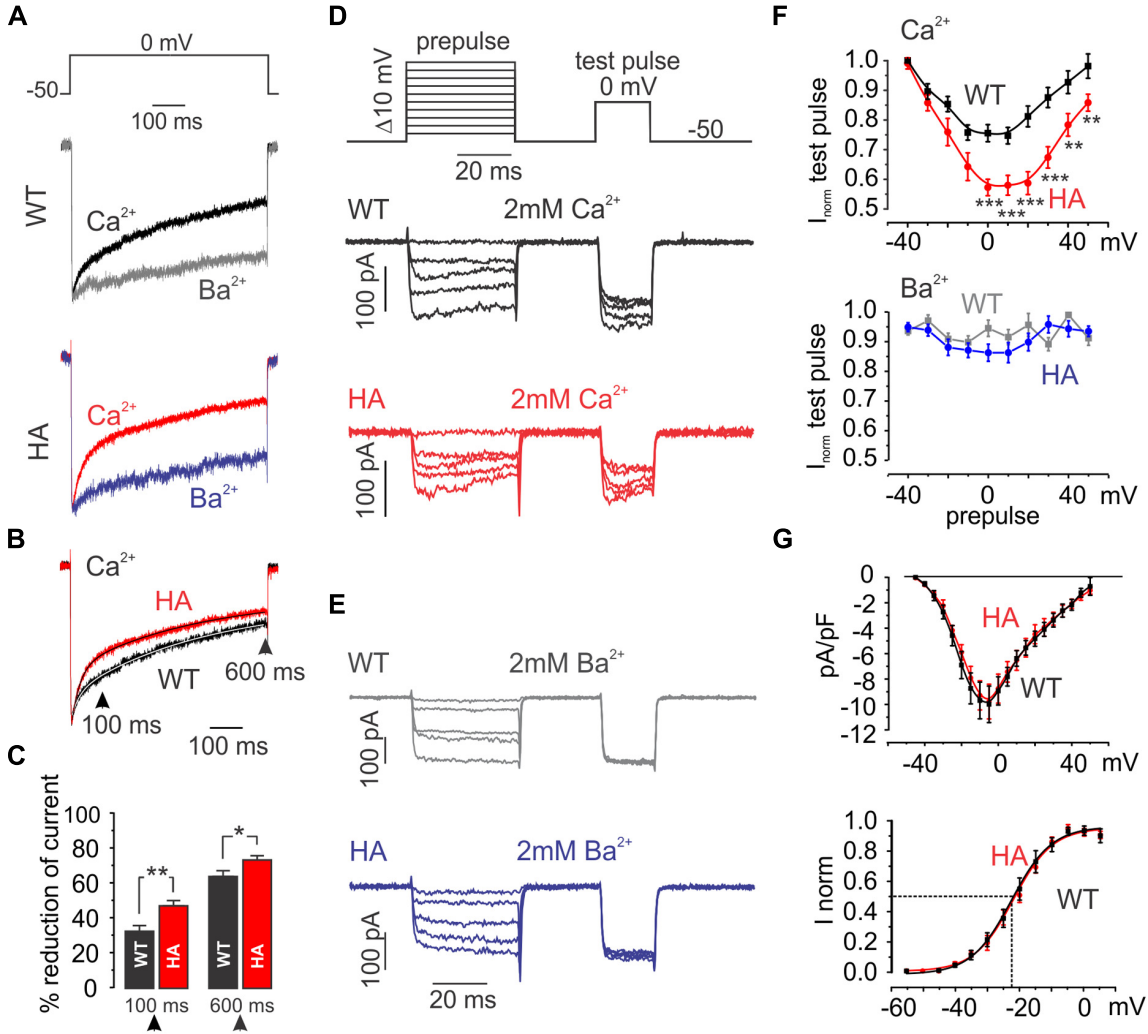
**L-type currents in MCCs from WT and Cav1.3DCRD^HA/HA^ mice. (A)** Averaged L-type currents of WT (*n* = 23) and Cav1.3DCRD^HA/HA^ (HA, *n* = 27) MCCs measured in 2 mM Ca^2+^ (black and red) or 2 mM Ba^2+^ (gray and blue) triggered by the protocol shown on top. L-type currents were obtained by subtraction of nifedipine (3 μM) resistant current from control traces and normalized to the peak. **(B)** Superimposed L-type currents obtained in 2 mM Ca^2+^ from WT and Cav1.3DCRD^HA/HA^ MCCs. Double exponential fits of the averaged traces (continuous curves within traces) revealed the following parameters: A_fast_ = -0.14, A_slow_ = -0.57, τ_fast_ = 23.8 ms, τ_slow_ = 397.7 ms and C = -0.25 for WT and A_fast_ = -0.34, A_slow_ = -0.46, τ_fast_ = 24.9 ms, τ_slow_ = 382.9 ms and C = -0.20 for DCRD^HA/HA^ MCC L-type currents. **(C)** Percent inactivation of I_Ca_ after 100 and 600 ms depolarization from V_h_ = -50 to 0 mV for WT (black, *n* = 23) and DCRD^HA/HA^ (red, *n* = 27) MCCs (^∗^*p* < 0.05; ^∗∗^*p* < 0.01, Student’s *t*-test). **(D)** Double-pulse protocol used to evaluate Ca^2+^-dependent inactivation (CDI) and representative traces in 2 mM Ca^2+^ of WT (black) and Cav1.3DCRD^HA/HA^ (red) MCCs. CDI induced by 40-ms depolarizations to different voltages was evaluated by a test pulse to 0 mV. Test pulse currents were normalized to maximal current amplitude obtained after prepulses to -40 mV during which only a very small fraction of current was activated **(G)**. **(E)** Protocol as in **(D)** to compare inactivation in 2 mM Ba^2+^ between genotypes. As for **(A–C)**, L-type currents were obtained by subtraction of nifedipine (3 μM) – resistant currents from total current. **(F)** Top: test pulse current peaks plotted against pre-pulse conditioning voltage. Currents were normalized against maximal peak current for WT (*n* = 26, black squares) Cav1.3DCRD^HA/HA^ (*n* = 22, red dots) MCCs. Bottom: same analysis but using Ba^2+^ as charge carrier for WT (*n* = 7, gray squares) and Cav1.3DCRD^HA/HA^ (*n* = 8, blue circles) MCCs (^∗∗∗^*p* < 0.001; ^∗∗^*p* < 0.01, two-way ANOVA followed by Bonferroni post-test). **(G)** Top: current–voltage relationship of WT (black squares) and Cav1.3DCRD^HA/HA^ (red dots) MCCs. Bottom: normalized conductance fit with a Boltzmann function: V_0.5_ = -23.3 mV, k = 6.5 mV for WT and V_0.5_ = -21.9 mV, k = 6.3 mV for Cav1.3DCRD^HA/HA^ MCCs.

From studies in recombinant channels the increased current amplitude in mutant IHCs was predicted whereas the slowing of CDI was not. We therefore also studied the consequences of CTM inhibition in MCCs. In MCCs robust CDI of L-type currents indicates no detectable effects of inhibitory CaBPs (Marcantoni et al., 2010). In WT MCCs about 50% of the I_Ca_ is L-type (i.e., nifedipine-sensitive) and equally carried by Cav1.2 and Cav1.3 (Marcantoni et al., 2010). The remainder of the current is non-L-type (P/Q-, N-, and R-type). At holding potentials of -50 mV (near MCC resting potential) 3 μM nifedipine fully blocks L-type currents (Mahapatra et al., 2011). By subtracting the nifedipine-resistant component from total I_Ca_, L-type currents (I_Ca,L_) can be isolated (Marcantoni et al., 2010). In 2 mM Ca^2+^ I_Ca,L_ inactivated with a fast and a slow component during 600-ms pulses to 0 mV (**Figure 9A**). Inactivation of I_Ba_ (2 mM Ba^2+^, VDI) was much slower revealing robust CDI. Cav1.3DCRD^HA/HA^ MCCs showed no differences in VDI compared to WT but significantly faster inactivation was observed for I_Ca,L_ (**Figures 9A,F**). This is evident when normalized I_Ca,L_ recordings from WT and Cav1.3DCRD^HA/HA^ MCCs were superimposed (**Figure 9B**). In Cav1.3DCRD^HA/HA^ MCCs the degree of I_Ca,L_ inactivation was larger both after 100 ms (WT: 32.5 ± 3.2%, Cav1.3DCRD^HA/HA^ 47 ± 3%, *p* < 0.01, *n* = 27) and 600 ms (63.7 ± 3.3 vs. 73 ± 2.4%, *p* < 0.05, *n* = 23) (**Figure 9C**). Double-exponential fits of the averaged normalized traces indicated the presence of a fast and slow inactivating component with similar time constants in MCCs of both genotypes (see legend to **Figure 9B**). However, in mutant MCCs the fractional contribution of τ_fast_ was about twofold larger (A_fast_: WT: 0.14, Cav1.3DCRD^HA/HA^: 0.34) with a corresponding reduction of the contribution of the slow (A_slow_) and non-inactivating component (C) (**Figure 9B** legend). Similar significant differences were obtained when individual L-type currents were fitted (A_fast_: WT (*n* = 16): -0.24 ± 0.04, Cav1.3DCRD^HA/HA^ (*n* = 22): -0.36 ± 0.04, *p* < 0.05; A_slow_: WT: -0.50 ± 0.06, Cav1.3DCRD^HA/HA^: -0.35 ± 0.04, *p* < 0.05; non-inactivating: WT: -0.26 ± 0.03, Cav1.3DCRD^HA/HA^: -0.29 ± 0.03).

We also quantified the voltage-dependence of CDI with a double-pulse protocol (**Figures 9D,F**). CDI was again significantly different over a large voltage range and revealed the CDI-typical U-shaped inactivation characteristics (Ben-Johny and Yue, 2014) absent in I_Ba_ recordings (**Figures 9E,F**). Since current densities between both WT and DCRD^HA/HA^ MCCs had comparable amplitude and voltage-dependence of activation (**Figure 9G**) we conclude that the observed effect on inactivation is inherent to a difference in CaM-dependent CDI. Since the current–voltage relationships comprise both Cav1.2 and Cav1.3 (or Cav1.3DCRD^HA/HA^) components effects of the mutation on the V_0.5_ cannot be reliably determined.

**FIGURE 10:**
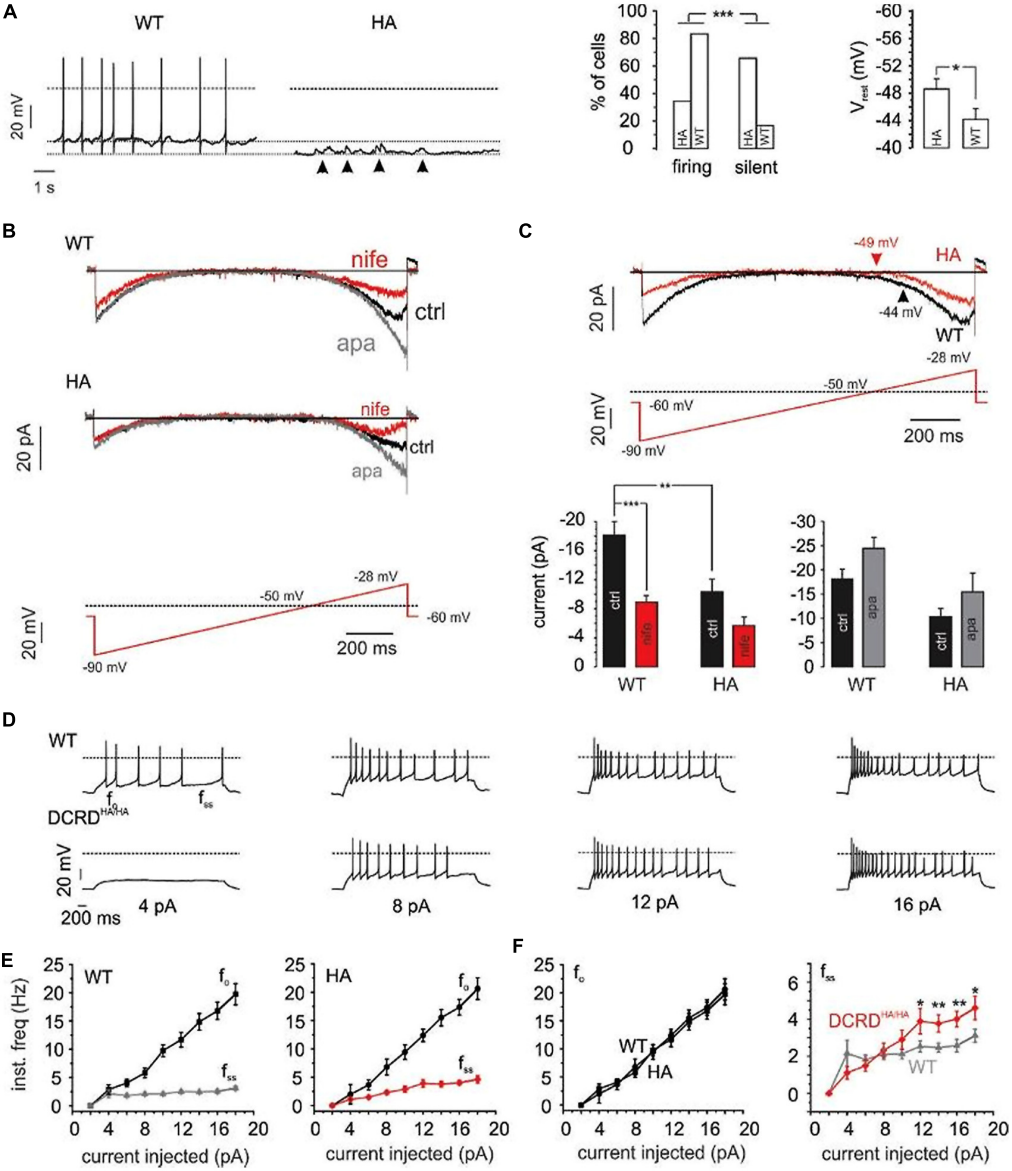
**Firing properties of WT – vs. DCRD^HA/HA^ MCCs. (A)** Left: current-clamp traces of representative WT (black) and Cav1.3DCRD^HA/HA^ (red) MCCs without current injection. Dashed lines indicate baseline, dotted line indicates 0 mV. Right: percent of firing vs. silent cells and comparison of V_rest_ for WT (*n* = 18) and Cav1.3DCRD^HA/HA^ (HA, *n* = 32) MCCs. Significance testing on categorical data was performed by RxC contingency tables and a chi–square test (^∗∗∗^*p* < 0.001) while a Student’s *t*-test was used for V_rest_ (^∗^*p* < 0.05). **(B)** Averaged Na^+^, K^+^, and Ca^2+^-currents of WT (*n* = 9) and Cav1.3DCRD^HA/HA^ (*n* = 7) MCCs to the illustrated slow ramp protocol in the absence (black, ctrl) or presence of nifedipine (3 μM, red) and during SK channel block by apamin (200 nM, gray). **(C)** Top: overlay of WT (black) and Cav1.3DCRD^HA/HA^ averaged currents (red) elicited by the indicated ramp protocol. Arrows indicate the mean V_rest_ of both cell populations. Bottom: statistics for peak inward currents at about -25 mV triggered by the ramp protocol of control traces (black), during nifedipine (red) or SK channel block by apamin (gray) for WT (*n* = 9) and Cav1.3DCRD^HA/HA^ (*n* = 7) MCCs (^∗∗^*p* < 0.01, ^∗∗∗^*p* < 0.001, one–way ANOVA followed by a Bonferroni *post hoc* analysis). **(D)** Representative current-clamp traces at increasing current injections from *V*_h_ = -70 mV from WT (black) and Cav1.3DCRD^HA/HA^ (red) MCCs. **(E)** Graphical representation of f_o_ and f_ss_ for WT (*n* = 15) and Cav1.3DCRD^HA/HA^ (*n* = 16) MCCs with increasing current injections. f_o_, is the first inter-spike interval frequency and f_ss_, is the last inter-spike interval frequency. **(F)** Overlay of f_o_ and f_ss_ from **(E)** to highlight differences (^∗^*p* < 0.05, ^∗∗^*p* < 0.01, paired Student’s *t*-test).

We have recently demonstrated a critical role of Cav1.3 activity for the generation of spontaneous action potentials (APs) in MCCs (Vandael et al., 2010, 2012). Given the increased rate of inactivation of Cav1.3 channels in Cav1.3DCRD^HA/HA^ MCCs we tested if the integrity of the CTM is important for MCC firing properties. In accordance with our previous findings (Vandael et al., 2012) 15 out of 18 WT MCCs (83%) fired spontaneously in current-clamp with no current injection. In contrast, only 11 out of 32 Cav1.3DCRD^HA/HA^ MCCs (34%) showed spontaneous APs (*p* < 0.001, **Figure 10A**). The resting membrane potential (V_rest_) was significantly hyperpolarized by 4.4 mV in Cav1.3DCRD^HA/HA^ MCCs as compared to WT (*p* < 0.05; **Figure 10A**). Quiescent, spontaneous sub-threshold oscillations of 4–6 mV lasting 0.2–0.5 s were observed in Cav1.3DCRD^HA/HA^ MCCs (arrows in **Figure 10A**), indicating the tendency of these cells to depolarize without reaching the threshold of AP firing. This is most likely due the more pronounced CDI decreasing the contribution of subthreshold Cav1.3 channel activity to the net inward current driving AP firing. In spontaneously firing cells the AP frequency was not different between both groups (1.4 ± 0.5 Hz for Cav1.3DCRD^HA/HA^, 1.5 ± 0.3 Hz for WT) and neither were other spike parameters.

Next we used slow-ramp voltage-clamp commands (27 mV/s, -90 to -28 mV near spike threshold) to test for the size and time course of pacemaker currents (Ca^2+^, K^+^, Na^+^). These parameters were selected since control MCCs depolarize from about -55 to -28 mV in 1 s (1 Hz) during pacemaking. WT MCCs (*n* = 9) showed significantly larger inward currents than Cav1.3DCRD^HA/HA^ MCCs (*p* < 0.01; *n* = 9; **Figures 10B,C**). These currents were effectively blocked by 3 μM nifedipine (50% for WT and 45% for DCRD^HA/HA^ MCCs) indicating a major contribution by LTCCs (**Figure 10B**). I_Ca_ activation was shifted to more positive voltages in Cav1.3DCRD^HA/HA^ MCCs (red trace in **Figure 10C**). At pacemaker potentials, Cav1.3 channels are known to activate SK-channels which dampen pacemaking and reduce the firing frequency in MCCs (Vandael et al., 2012). SK currents are able to contribute to outward currents at relatively negative membrane potentials. We thus investigated the contribution of SK currents during the pacemaker cycle by applying 200 nM apamin. During slow ramp depolarizations SK currents in Cav1.3DCRD^HA/HA^ MCCs were smaller than in WT (difference not significant, **Figure 10C** bottom-right). The same trend was also observed when considering the apamin- and nifedipine-sensitive outward tail current that follows the slow ramp (**Figures 10B,C**). In both cases, the WT and Cav1.3DCRD^HA/HA^ control traces resulted in a net inward current that indicates a principal contribution of Ca^2+^ channels to the overall pacemaking current (**Figure 10C**). Less Ca^2+^-influx at rest due to more pronounced CDI can explain the observed hyperpolarization of V_rest_ and reduced number of spontaneously firing DCRD^HA/HA^ MCCs.

Upon injection of current pulses of increasing intensity (2–18 pA) from a V_h_ of -70 mV (**Figure 10D**), 4 out of 16 Cav1.3DCRD^HA/HA^ cells and 4 out of 16 WT cells started spiking during 4 pA current injections. The firing frequency at the onset (f_o_) and steady-state (f_ss_) increased with current intensity in WT and Cav1.3DCRD^HA/HA^ MCCs, suggesting spike frequency adaptation in both genotypes (**Figure 10E**; Vandael et al., 2012). There was no significant difference between f_o_ at any given different current intensity, while at higher current intensities f_ss_ was significantly higher in Cav1.3DCRD^HA/HA^ than in WT MCCs (**Figure 10F**). The origin of this phenomenon is likely an altered Cav1.3/SK coupling mechanism. The accelerated CDI of Cav1.3DCRD^HA/HA^ channels is expected to recruit less SK currents during repetitive firing. This in turn would reduce the mean outward current passing during the interspike interval with consequent increases in the rate of firing (f_ss_).

**FIGURE 11:**
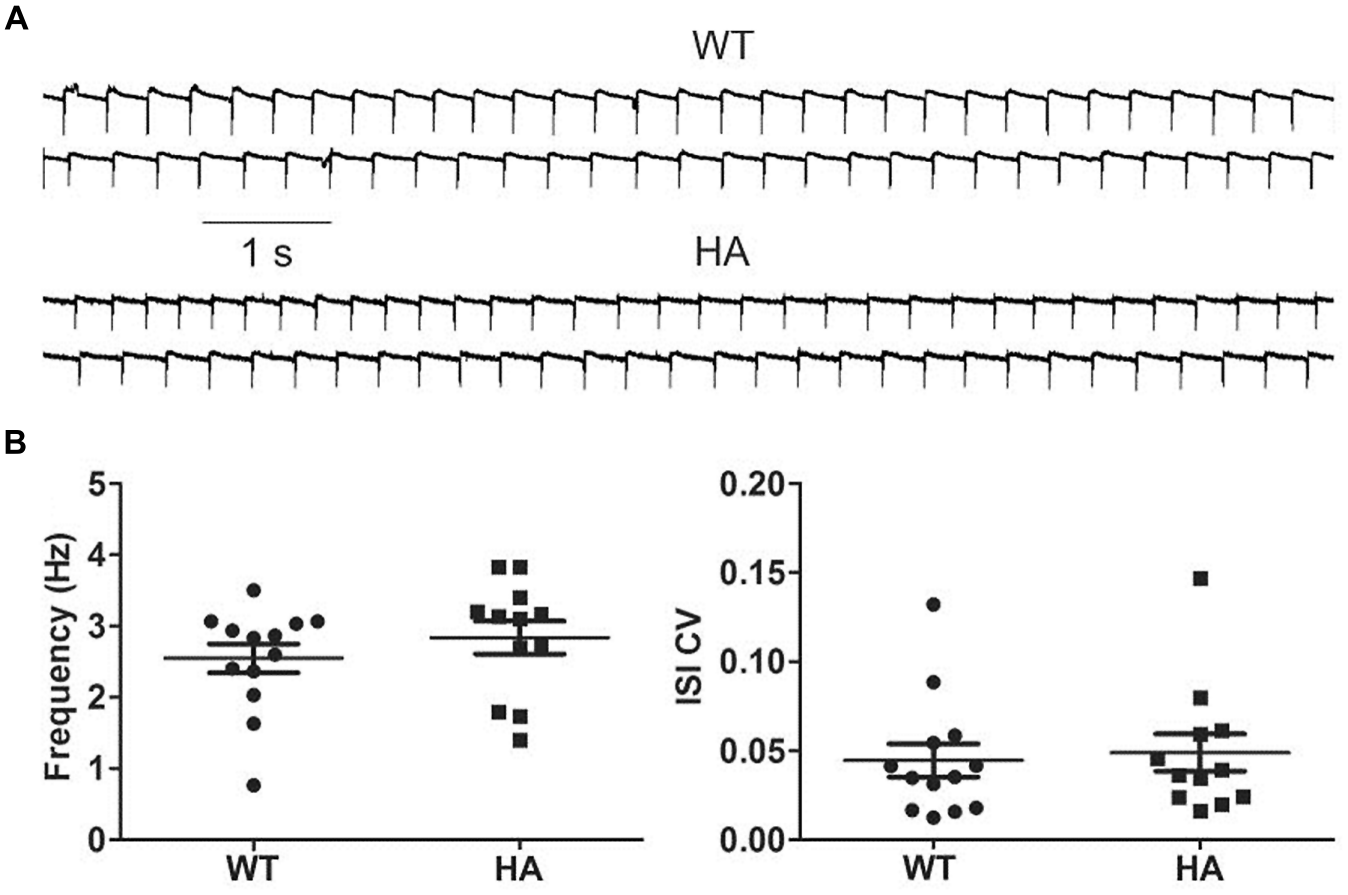
**Spontaneous pacemaking in WT and Cav1.3DCRD^HA/HA^ SN DA neurons. (A)** Cav1.3DCRD^HA/HA^ (HA) and WT neurons were held in cell-attached mode (sample traces). **(B)** They typically fired continuously with no statistical differences in frequencies (WT: 2.5 ± 0.2 Hz, *n* = 13; HA: 2.8 ± 0.2 Hz, *n* = 12; mean ± SE) and the coefficient of variation of the inter-spike interval (ISI CV) (0.045 ± 0.009 for WT and 0.048 ± 0.010 for HA). Putative dopamine neurons in the substantia nigra pars compacta were identified based of their position within the slice, their large size and the presence of I_H_-mediated hyperpolarization upon current injection (>100 pA). Dopamine neurons were dialyzed with biocytin. After tissue fixation, their identity was verified by streptavidin and tyrosine hydroxylase (TH) co-staining.

Because a role of Cav1.3 has also been implicated in the pacemaking of substantia nigra dopamine neurons (SN DA), and both long and short Cav1.3 channel variants are expressed in these cells (Olson et al., 2005), we also tested if spontaneous pacemaking is affected in these neurons. However, no difference was observed between SN DA neurons in acute brain slices from Cav1.3DCRD^HA/HA^ mice and their WT littermates (**Figure 11**).

## Discussion

We generated a novel Cav1.3 mouse model that allowed us to specifically study the role of a C-terminal automodulatory domain previously discovered in recombinant expression systems (Singh et al., 2008; Tan et al., 2011) but with unknown function on I_Ca_ in native cells and *in vivo* functions. By introducing an HA-antibody tag we disrupted CTM function *in vivo* and thus induced the gating behavior of short Cav1.3 splice variants also in the long Cav1.3 isoform. Study of the mutant channels in several tissues provided us with novel insights into the physiological role of this modulatory mechanism. We show that it is required for fine-tuning of the activity of Cav1.3 channels not only in recombinant systems but also in their native environment. The functional impact of the CTM varies in a cell-type specific manner: it supports a fast, Ca^2+^-dependent component of channel inactivation in IHCs but suppresses CDI in MCCs. Although Cav1.3 LTCC currents account for only about 25% of the total I_Ca_ in MCCs (Marcantoni et al., 2010), interruption of CTM function caused a profound change in their electrical activity. This was reflected by a more negative resting potential, reduced Ca^2+^-influx during spontaneous pacemaking, less spontaneous activity and less spike frequency adaptation. These changes could be explained by increased CDI in mutant channels with reduced signaling to SK-channels (Vandael et al., 2012).

Several groups have revealed important molecular details of CaM-regulation of VGCCs including Cav1.3 and Cav1.2 LTCCs (Christel and Lee, 2012; Ben-Johny and Yue, 2014 employing recombinant channels expressed in mammalian cells (mainly HEK-293). This has not only provided critical insight into CaM-mediated CDI and Ca^2+^-dependent facilitation, but also into mechanisms that adjust the strength of CaM modulation even further. By reducing the affinity for apoCaM pre-association with the proximal C-terminus of the α1-subunit CaM regulation becomes tunable by ambient CaM concentrations (Bazzazi et al., 2013). The CTM studied here represents such a mechanism, in addition to RNA editing (Huang et al., 2012). Moreover, CaBPs which do not mediate CDI can competitively (Oz et al., 2011; Findeisen et al., 2013) and/or allosterically affect CaM binding (Yang et al., 2014) and completely remove CDI when overexpressed with Cav1.3. However, much less is known about how this complex modulation affects Cav1.3 channel activity in excitable cells that differ with respect to intracellular CaM and CaBP concentrations, RNA editing, and the relative abundance of long and short Cav1.3 splice variants. Our mouse model allowed us to directly investigate this question. By selecting two different cell types, IHCs and MCCs, in which Cav1.3 current components can be measured separate from other L- and non-L-type currents, we demonstrate that the modulatory effects of the CTM in Cav1.3 are cell-type dependent. From the analysis of recombinant mCav1.3_L_-HA channels (**Figure 1**) and from previous work (Bock et al., 2011; Tan et al., 2011) we expected that disabling CTM activity would (i) enhance I_Ca_ amplitude (due to higher open probability, Bock et al., 2011); (ii) facilitate channel activation at more negative voltages and (iii) permit more pronounced CDI (Ben-Johny and Yue, 2014; Lieb et al., 2014). L-type I_Ca_ in MCCs exhibit CDI (**Figure 9A**), and the expected CDI increase was observed despite the fact that Cav1.3 accounts for only about 50% of the L-type current and about 25% of total I_Ca_ (Marcantoni et al., 2010). In contrast, IHCs are known to display very weak, but measurable, CDI due to the abundant expression of CaBPs. We found that this weak CDI was even reduced in Cav1.3DCRD^HA/HA^ IHCs. Whereas the increase in current density is expected for channel gating unopposed by the CTM, decreased CDI is contrary to predictions from HEK-293 cell-expressed channels and our findings in MCCs. This clearly demonstrates that the CTM can even promote CDI in a specific cellular environment. An obvious explanation for this unexpected finding is that in IHCs CaBPs and CaM compete for modulation of CDI (Oz et al., 2011; Findeisen et al., 2013; Yang et al., 2014). Removal of the functional CTM therefore may not only facilitate CaM but also CaBP interaction with the channel. We therefore hypothesize that the absence of the CTM favors CaBP binding relative to CaM leading to the observed reduction of CDI. Testing this hypothesis in heterologous expression systems will be challenging due to the toxicity of CaBPs when co-expressed with Cav1.3 (Yang et al., 2014) and the need to demonstrate graded and quantitatively different effects of CaBPs (in particular CaBP1 and CaBP2, the main CaBPs in IHCs, Cui et al., 2007; Schrauwen et al., 2012) on CDI of WT and mCav1.3_L_-HA or C-terminally short splice variants.

We also took advantage of the presence of an HA-antibody tag to demonstrate that long Cav1.3 isoforms are an intrinsic part of all Cav1.3 clusters at ribbon synapses in adult IHCs. In both inner and outer hair cells our nested PCR data also revealed the expression of Cav1.3_43S_, the major short Cav1.3 α1-subunit splice variant expressed in the brain (Bock et al., 2011; Tan et al., 2011). The Cav1.3DCRD^HA/HA^ mouse model will be a valuable tool to quantify the relative abundance of long and short Cav1.3 splice variants on the protein level in IHCs and other tissues, as reported here for brain. Western blot analysis using anti-HA as well as N- and C-terminal anti-Cav1.3 antibodies allowed us to confirm the presence of two different size α1-subunit species with molecular masses differing by 54 kDa. The larger band must contain the exon 49-encoded C-terminal end that also harbors the HA-antibody tag in Cav1.3DCRD^HA/HA^ mice. In WT Cav1.3 it must therefore represent the protein responsible for “long” gating behavior *in vivo*. The absence of smaller HA-tagged peptides argues against the existence of significant “midchannel” proteolysis of Cav1.3 (as recently postulated for Cav1.2; Michailidis et al., 2014) and also against the presence of stable C-terminal peptides that could serve as transcriptional regulators. For Cav1.2 such a peptide was found to act as a transcriptional regulator in brain (Gomez-Ospina et al., 2006) and, non-covalently attached to cardiac Cav1.2 α1, is required for normal regulation by cAMP-dependent protein kinase (Fu et al., 2013). Post-translational C-terminal proteolytic cleavage of Cav1.3 α1 has recently also been postulated for cardiac tissue (Lu et al., 2015). However, neither our C-terminal antibody in WT nor anti-HA antibodies in Cav1.3DCRD^HA/HA^ mice detected such fragments in mouse brain.

Our findings also have important implications for understanding the pathophysiological role of Cav1.3 channels. As outlined in the introduction, distinct changes in Cav1.3 channel gating by single missense mutations can cause human disease. Gating changes permitting enhanced Ca^2+^ inward current through Cav1.3 were not only identified as cause for excessive aldosterone secretion in adrenal adenomas (Azizan et al., 2013) but also as cause for PASNA, a severe human congenital disease (Scholl et al., 2013) and as high risk *de novo* mutations for autism with intellectual disability (Pinggera et al., 2015). As our mouse model also introduces gating changes (e.g., steeper voltage-dependence, enhanced open probability) that could enhance Cav1.3 mediated Ca^2+^-influx in neurons, it is ideally suited to address the question if dysregulation of Cav1.3 underlies neuropsychiatric phenotypes in future behavioral studies. Interestingly, two rare genetic variants were reported (rs41276455, rs150313433) that both neutralize the negative charge of Asp-2117 (NCBI reference NP_000711.1), one of the negative charges in the DEME sequence (**Figure 1D**) which we have shown to be required for interaction with PCRD and formation of a functional CTM (Singh et al., 2008). Although these variants have not yet been investigated for association with disease risk, our functional data in mice suggest this possibility. Our work emphasizes the importance of efforts to identify the *in vivo* functional relevance of modulatory domains in Cav1.3 LTCCs channels as shown here for the CTM.

## Conflict of Interest Statement

The authors declare that the research was conducted in the absence of any commercial or financial relationships that could be construed as a potential conflict of interest.
